# Correlates of physical activity and sedentary behavior among cancer survivors and cancer-free women: The Women’s Health Accelerometry Collaboration

**DOI:** 10.1371/journal.pone.0301233

**Published:** 2024-04-04

**Authors:** Samantha Schilsky, Annie Green Howard, Christopher C. Moore, Carmen C. Cuthbertson, Humberto Parada, I-Min Lee, Chongzhi Di, Michael J. LaMonte, Julie E. Buring, Eric J. Shiroma, Andrea Z. LaCroix, Kelly R. Evenson

**Affiliations:** 1 Department of Epidemiology, Gillings School of Global Public Health, University of North Carolina Chapel Hill, Chapel Hill, North Carolina, United States of America; 2 Department of Biostatistics, Gillings School of Global Public Health, University of North Carolina Chapel Hill, Chapel Hill, North Carolina, United States of America; 3 Carolina Population Center, Gillings School of Global Public Health, University of North Carolina Chapel Hill, Chapel Hill, North Carolina, United States of America; 4 Department of Health Education and Promotion, East Carolina University, Greenville, North Carolina, United States of America; 5 Division of Epidemiology and Biostatistics, School of Public Health, San Diego State University, San Diego, California, United States of America; 6 UC San Diego Health Moores Cancer Center, La Jolla, California, United States of America; 7 Division of Preventive Medicine, Department of Medicine, Brigham and Women’s Hospital, Harvard Medical School, Boston, Massachusetts, United States of America; 8 Department of Epidemiology, Harvard TH Chan School of Public Health, Boston, Massachusetts, United States of America; 9 Biostatistics Program, Public Health Sciences Division, Fred Hutchinson Cancer Center, Seattle, Washington, United States of America; 10 Department of Epidemiology and Environmental Health, University of Buffalo, Buffalo, New York, United States of America; 11 Clinical Applications and Prevention Branch, National Institutes of Health, National Heart Lung Blood Institute, Bethesda, Maryland, United States of America; 12 Herbert Wertheim School of Public Health and Human Longevity Science, University of California, San Diego, La Jolla, California, United States of America; Saga University, JAPAN

## Abstract

**Background:**

Describing correlates of physical activity (PA) and sedentary behavior (SB) among postmenopausal cancer survivors can help identify risk profiles and can be used to support development of targeted interventions to improve PA and reduce SB in this population.

**Objective:**

To describe PA/SB and identify correlates of PA/SB among cancer and cancer-free post-menopausal women.

**Methods:**

Women from the Women’s Health Study (N = 16,629) and Women’s Health Initiative/Objective Physical Activity and Cardiovascular Health Study (N = 6,079) were asked to wear an accelerometer on the hip for 7 days. Multiple mixed-effects linear regression models were used to identify sociodemographic-, health-, and chronic condition-related correlates (independent variables) associated with PA and SB (dependent variables) among women with (n = 2,554) and without (n = 20,154) a history of cancer. All correlates were mutually adjusted for each other.

**Results:**

In unadjusted analyses, women with a history of cancer took fewer mean daily steps (4,572 (standard deviation 2557) vs 5,029 (2679) steps/day) and had lower mean moderate-to-vigorous PA (74.9 (45.0) vs. 81.6 (46.7) minutes/day) than cancer-free women. In adjusted analyses, for cancer and cancer-free women, age, diabetes, overweight, and obesity were inversely associated with all metrics of PA (average vector magnitude, time in moderate-to-vigorous PA, step volume, time at ≥40 steps/minutes, and peak 30-minute step cadence). In unadjusted analyses, mean SB was similar for those with and without cancer (529.7 (98.1) vs. 521.7 (101.2) minutes/day). In adjusted analyses, for cancer and cancer-free women, age, diabetes, cardiovascular disease, current smoking, overweight, and obesity were positive correlates of SB, while Black or Hispanic race/ethnicity, weekly/daily alcohol intake, and excellent/very good/good self-rated health were inverse correlates of SB.

**Conclusion:**

Several sociodemographic, health, and chronic conditions were correlates of PA/SB for postmenopausal women with and without cancer. Future studies should examine longitudinal relationships to gain insight into potential determinants of PA/SB.

## Introduction

For cancer survivors, maintaining healthy lifestyle behaviors, including physical activity (PA), into older adulthood is important for preventing cancer recurrence and mortality [1]. PA, defined as any bodily movement produced by skeletal muscles resulting in energy expenditure, is a beneficial behavior for cancer survivors [2, 3] and is associated with a lower risk of breast, prostate, and colorectal cancer recurrence and mortality [4, 5]. Sedentary behavior (SB) is any waking behavior in a sitting, reclining, or lying posture that requires less than 1.5 metabolic equivalents [3]. SB is associated with weight gain, and increased risk of cardiovascular disease (CVD) and mortality among colorectal cancer survivors [6]. With an estimated 8.3 million male and 9.7 million female United States’ cancer survivors in 2022 and a projected national increase in prevalence in the coming years, elucidating correlates of health behaviors, such as PA and SB, for cancer survivors is an important public health priority [1, 7].

Adult PA and SB differ among those with and without a history of cancer [8–10]. Differences in PA and SB may be due to cancer treatment and respective adverse effects which may impact survivor engagement in PA. Previous studies demonstrated failure to return to pre-cancer diagnosis levels of PA during and following treatment [11–15]. As cancers and subsequent treatments are heterogeneous, PA and SB may differ by primary cancer diagnosis due to type and longevity of cancer treatment.

Previous studies have examined correlates of accelerometer-assessed PA and SB in survivors diagnosed with non-Hodgkins lymphoma [16], lung [17], breast [18], and colon cancer [19]. Expanding this body of research to additional cancer types can further our understanding of how PA and SB can be leveraged to enhance cancer survivor’s prognosis and quality of life.

Among women in the Women’s Health Accelerometry Collaboration (WHAC), we described accelerometer-measured PA/SB and identified correlates of PA/SB separately for those with and without a history of cancer. For women with cancer, we also described PA/SB and identified correlates of PA/SB by common cancer types. Our work was hypothesis-generating, given the limited literature on the topic.

## Materials and methods

### Study design

The WHAC pooled data from the Women’s Health Initiative Objective Physical Activity and Cardiovascular Health (WHI/OPACH) ancillary study and the Women’s Health Study (WHS). These studies were comprised of women previously enrolled in the WHI Clinical Trials, the WHI Observational Study [20], or the WHS randomized trial [21, 22]. The WHI Clinical Trials and the Observational Study (1993–1998) enrolled 161,808 postmenopausal women ages 50 to 79 years of age into a randomized clinical trials component or a prospective observational study at 40 clinical centers across the United States [20, 23]. Among these women, 7,048 had accelerometer data captured in an additional ancillary study, the WHI/OPACH study [24]. The WHS was a randomized trial (1992–2004) testing aspirin, beta-carotene, and vitamin E for preventing CVD and cancer among 39,876 healthy United States’ women 45 years of age or older [22, 25, 26]. After trial completion, women who were willing continued to participate in an observational study that captured lifestyle and health habits as well as medical history annually. In 2011–2015, 18,289 women ages 62 to 89 years of age, who were able to walk outside of their homes unassisted, participated in an ancillary study that collected accelerometer assessments of PA and SB [27].

The WHI/OPACH and WHS had similar data collection methodologies and utilized the same accelerometer, as detailed elsewhere [28]. Study protocols were approved by the Fred Hutchinson Cancer Research Center Institutional Review Board and the Brigham and Women’s Hospital Institutional Review Board for the WHI/OPACH and WHS study, respectively. All women provided written informed consent or consented by telephone prior to data collection.

### Cancer diagnosis

Women who reported a diagnosis of cancer prior to accelerometer data collection were considered cancer survivors. Annual mailings were sent to women that inquired regarding cancer diagnoses, hospitalizations, and other health history items. Medical records were obtained for all women who self-reported a diagnosis of any cancer (except non-melanoma skin cancer) [29]. Physician adjudicators coded cancers with the International Classification of Diseases for Oncology. All participants were classified as either cancer-free (n = 20,154) or cancer survivors (n = 2,554). Cancer survivors were classified by cancer subtypes. Women were categorized as having multiple cancer types if diagnosed with more than one primary cancer. We focused on the five most common cancer subtypes in this cohort including breast, endometrial, colon, melanoma skin, and lung cancers. Date of diagnosis was defined as the earliest occurrence of histological confirmation or date of the first hospitalization for each cancer type [30]. Time since cancer diagnosis was derived as the date from cancer diagnosis to date of accelerometer data collection and reported by cancer type.

### Physical activity and sedentary behavior

An ActiGraph GT3X+ accelerometer (Pensacola, Florida, US) was used by both the WHI/OPACH and WHS cohort studies. Women were asked to wear the accelerometer on their right hip for seven consecutive days during waking hours. Women in the WHI/OPACH study were asked to wear the accelerometer during non-waking hours as well. Data collected during non-waking hours in the WHI/OPACH cohort were removed for congruence between the cohorts. The accelerometer recorded at 30 Hz and was aggregated using ActiLife software (version 6) to counts per 15-second epochs using the normal filter. Non-wear time was identified using the validated Choi algorithm [31], defined as an interval of at least 90 consecutive minutes of zero vector magnitude (VM) counts/minute, with allowance of up to 2 minutes of nonzero VM counts if no nonzero counts were detected during both the 30 minutes upstream and downstream from that interval. Adherence was defined as ≥4 days/week and ≥10 hour/day of accelerometer wear.

From the accelerometer data averaging over adherent days, VM/15-second epoch (VM/15-s) cutpoints were derived for time spent in sedentary (0–18 VM/15-s), low light (19–225 VM/15-s), high light (226–518 VM/15-s), and moderate to vigorous PA (MVPA, ≥519 VM/15-s). These thresholds were based on a calibration study conducted among women of similar ages, with PA intensity defined as low light (1.6–2.2 metabolic equivalents (METS)), high light 2.3–2.9 METS, and MVPA (≥3.0 METS) [32]. Light intensity was differentiated because of the large amount of time spent in this category. We also derived average total volume, summarized as average VM/15-s, that was not based on cutpoints.

From the accelerometer data, the following step metrics were derived: average total daily steps (steps/minute), average peak 30-minute cadence (defined as the highest steps/minute for 30 minutes of the day and not necessarily consecutive minutes), and time spent in purposeful steps and faster ambulation (≥40 steps/minute) [33]. Around the same time as accelerometer data collection, women were also asked to self-report their walking pace via questionnaire, with details provided elsewhere [28].

### Additional measurements

Additional measurements were assessed around the same time frame as accelerometer data collection. Potential correlates of PA and SB were grouped into sociodemographic, behavioral, and health-related characteristics and harmonized across the cohorts. The following sociodemographic characteristics were collected at study enrollment: age, race/ethnicity (White, Black, Hispanic, or other), and education (high school, general educational diploma (GED), or less; some college; or college graduates or more). Regularly completed mail-in questionnaires collected the following health behaviors and chronic health conditions: smoking (current, past, or never), alcohol intake (rarely, monthly, or weekly), menopausal hormone therapy use (yes or no), general health (excellent, very good, good, or fair/poor), history of CVD (yes or no), and history of diabetes (yes or no). Height and weight were self-reported in the WHS and measured in the WHI/OPACH study. Body mass index (BMI) was calculated as weight in kilograms divided by height in meters squared. BMI was categorized as underweight (<18.5 kg/m^2^), normal (18.5–24.9 kg/m^2^), overweight (25–29.9 kg/m^2^), or obese (>30 kg/m^2^).

### Exclusions

From the pooled sample from the WHS (N = 17,062) and WHI/OPACH cohort (N = 6,489), participants were excluded from this study if they had non-adherent accelerometer wear time (n = 575) (≥10 hours/day for ≥4 days) or were missing accelerometry data (n = 111). Participants were further excluded if they had cancer at the start of the WHS randomized trial (n = 16) or a cancer diagnosis within one year prior to the start of the accelerometer measurement in either cohort (n = 147). The final analytic sample included 22,708 women (16,629 from WHS and 6,079 from WHI/OPACH).

### Statistical analysis

Across cancer type, descriptive characteristics and PA metrics, self-reported walking pace, and SB were calculated as means and standard deviations for continuous variables and as counts and percentages for categorical variables. Multiple mixed-effects linear regression models with random intercepts for each cohort (WHS, WHI/OPACH) were used to estimate associations of potential correlates (independent variables) with PA metrics (dependent variables including average VM, time in MVPA, average total daily steps, time at ≥40 steps/minute, and peak 30-minute cadence) and SB. All regression models included the following independent variables: age, race/ethnicity, education, smoking status, alcohol intake, menopausal hormone therapy use, general health, history of CVD, history of diabetes, BMI, and accelerometer wear time. Models for associations among women with cancer and by cancer type were further adjusted for years since cancer diagnosis. In the analysis, all correlates were mutually adjusted for each other. A sensitivity analysis by cohort (i.e., separate for WHS and WHI/OPACH) was conducted separately for those with any cancer and those without cancer.

Multiple imputation by chained equations (MICE) with predictive mean matching was used to impute missing covariates. Smoking had the highest missing at 2.6% (n = 581). Imputed datasets were generated for each cohort and then combined for final analysis. We used 20 imputations, with 20 iterations, and included all potential correlates, PA/SB measures, and indicators for cancer type in the imputation model. A nominal α of 0.05 was used as a threshold for determining statistical significance. All models were calculated with SAS v. 9.4 (SAS Institute Inc., Cary, NC).

## Results

Women in the study had a mean (SD) age at accelerometry measurement of 73.4 (6.8) years (Table 1). Overall, 11.2% (n = 2554) of women had a prior cancer diagnosis (which included all cancers except non-melanoma skin cancer) with a mean of 8.7 (5.0) years since diagnosis. Breast cancer was the most prevalent cancer diagnosis (n = 1334), followed by endometrial (n = 240), colon (n = 175), melanoma (n = 169), and lung (n = 93) cancers.

**Table 1:** Characteristics of participants by cancer type; the Women’s Health Accelerometry Collaboration.

Characteristic	All (N = 22,708)	Cancer Free (N = 20,154)	Any Cancer (N = 2,554)	Breast cancer (N = 1,334)	Colon cancer (N = 175)	Endometrial cancer (N = 240)	Lung cancer (N = 93)	Melanoma skin cancer (N = 169)
Age; mean (SD)	73.4 (6.8)	73.3 (6.7)	74.4 (6.7)	73.9 (6.5)	76.7 (7.2)	73.6 (6.8)	76.2 (7.2)	73.8 (6.6)
Race/ethnicity								
White	83.1%	82.7%	85.6%	84.8%	81.1%	91.2%	83.9%	99.4%
Black	10.1%	10.2%	9.0%	9.2%	12.6%	4.6%	9.7%	0.0%
Hispanic	5.2%	5.3%	4.0%	4.3%	6.3%	2.5%	4.3%	0.6%
Other	1.7%	1.7%	1.4%	1.7%	0.0%	1.7%	2.2%	0.0%
Education level								
High school/GED or less	5.4%	5.4%	5.3%	4.6%	9.7%	2.9%	9.7%	1.8%
Some college	46.1%	46.2%	45.7%	45.4%	43.4%	46.7%	41.9%	54.4%
College graduate	47.0%	47.0%	48.0%	49.2%	45.1%	49.6%	47.3%	43.2%
Missing	1.40%	1.4%	0.9%	0.9%	1.7%	0.8%	1.1%	0.6%
Smoking								
Never	50.2%	50.5%	47.6%	47.8%	50.9%	50.8%	24.7%	50.9%
Past	43.9%	43.5%	47.7%	47.5%	43.4%	46.7%	74.2%	46.2%
Current	3.3%	3.4%	2.7%	2.8%	3.4%	0.8%	1.1%	2.4%
Missing	2.6%	2.6%	2.0%	1.9%	2.3%	1.7%	0.0%	0.6%
Alcohol intake								
Rarely	36.9%	36.8%	38.1%	36.7%	40.0%	40.8%	38.7%	33.1%
Monthly	15.5%	15.5%	15.7%	14.8%	19.4%	14.2%	15.1%	16.6%
Weekly	32.1%	32.3%	30.5%	31.3%	23.4%	34.2%	31.2%	31.4%
Daily	13.0%	12.9%	13.8%	15.4%	15.4%	9.6%	15.1%	18.3%
Missing	2.4%	2.4%	1.9%	1.8%	1.7%	1.2%	0.0%	0.6%
Current hormone therapy use								
No	92.0%	91.5%	96.2%	98.4%	92.6%	92.9%	94.6%	94.7%
Yes	7.9%	8.5%	3.8%	1.6%	7.4%	7.1%	5.4%	5.3%
Missing	0.03%	0.03%	0.0%	0.0%	0.0%	0.0%	0.0%	0.0%
Self-rated health								
Excellent	20.7%	21.5%	14.5%	13.9%	16.6%	15.0%	9.7%	20.1%
Very good	47.4%	47.9%	44.1%	45.7%	37.7%	41.2%	37.6%	49.1%
Good	27.3%	26.4%	34.1%	33.5%	36.6%	36.7%	39.8%	30.2%
Fair/poor	4.5%	4.1%	7.2%	6.8%	9.1%	7.1%	12.9%	0.6%
Missing	0.1%	0.1%	0.1%	0.1%	0.0%	0.0%	0.0%	0.0%
Body mass index								
Underweight	1.8%	1.8%	2.0%	1.6%	2.9%	2.1%	1.1%	0.0%
Normal weight	40.3%	40.3%	40.3%	40.9%	40.0%	36.7%	38.7%	43.2%
Overweight	34.1%	34.1%	34.3%	34.5%	32.0%	32.9%	39.8%	34.3%
Obese	22.1%	22.2%	21.7%	21.4%	23.4%	27.9%	18.3%	22.5%
Missing	1.7%	1.7%	1.8%	1.6%	1.7%	0.4%	2.2%	0.0%
History of cardiovascular disease								
No	94.2%	94.3%	93.7%	93.9%	95.4%	93.3%	90.3%	97.6%
Yes	5.8%	5.7%	6.3%	6.1%	4.6%	6.7%	9.7%	2.4%
History of diabetes								
No	88.0%	88.2%	85.8%	84.9%	81.7%	82.9%	89.2%	92.9%
Yes	12.0%	11.8%	14.2%	15.1%	18.3%	17.1%	10.8%	7.1%
Accelerometer wear time (minutes/day); mean (SD)	892.3 (75.9)	893.0 (76.2)	887.2 (73.2)	886.1 (70.7)	892.9 (71.5)	888.8 (79.3)	882.3 (71.7)	889.9 (76.7)
Time since cancer diagnosis (years) mean (SD)			8.7 (5.0)	8.9 (5.0)	9.2 (5.0)	9.2 (5.2)	7.4 (4.2)	9.0 (4.9)

Abbreviations: GED, general educational diploma; SD, standard deviation

Note: The frequencies and means reported in the table are not adjusted.

### Physical activity

Cancer survivors (e.g., “any cancer”) had lower average PA volumes and intensities (Table 2) and slower self-reported walking paces (Table 3) than cancer-free participants at the time of accelerometry data collection. Specifically, cancer survivors had lower weekly average time spent in MVPA (74.9 (SD = 45.0) minutes/day) than cancer-free women (81.6 (SD = 46.7)) and took fewer steps/day on average (4,572 (SD = 2,557)) than cancer-free women (5029 (SD = 2679) Table 2). Time spent in a stepping rate of ≥40 steps/minute as well as average peak 30-minute cadence were slightly lower for cancer survivors compared to cancer-free women. Time spent in high light PA and low light PA as well as time spent in 1–39 steps/minute was slightly lower for cancer survivors compared to cancer-free women. Fewer cancer survivors reported brisk or very brisk walking paces (Table 3).

**Table 2:** Mean (standard deviation) of physical activity and sedentary behavior metrics by cancer type; the Women’s Health Accelerometry Collaboration.

Characteristic	Cancer-Free n = 20,154	Any Cancer n = 2,554	Breast cancer n = 1,334	Colon cancer n = 175	Endometrial cancer n = 240	Lung cancer n = 93	Melanoma skin cancer n = 169
Sedentary behavior (min/day)	521.7 (101.2)	529.7 (98.1)	524.2 (98.4)	549.7 (97.3)	532.7 (101.4)	544.5 (108.5)	522.9 (94.9)
Average VM	134.9 (54.0)	127.0 (52.3)	130.1 (52.5)	114.8 (48.6)	129.5 (54.4)	116.4 (57.0)	134.6 (54.6)
Time in MVPA (min/day)	81.6 (46.7)	74.9 (45.0)	77.2 (45.3)	66.0 (41.4)	78.6 (47.0)	65.9 (47.5)	81.6 (47.0)
Time in low light (min/day)	182.6 (46.1)	178.6 (46.0)	179.6 (46.0)	177.5 (47.7)	174.0 (43.6)	174.7 (47.4)	177.8 (45.9)
Time in high light (min/day)	105.9 (33.3)	102.8 (33.6)	103.9 (33.2)	98.8 (33.4)	102.1 (36.4)	96.2 (37.3)	106.2 (33.1)
Total step volume	5029.2 (2679.2)	4572.0 (2557.4)	4686.5 (2520.3)	4050.1 (2452.7)	4696.2 (2795.0)	4208.0 (2817.2)	5014.3 (2817.7)
Time at 0 steps/min (min/day)	470.5 (101.8)	481.4 (98.8)	477.0 (99.5)	500.2 (99.6)	481.8 (98.8)	499.2 (107.5)	468.5 (93.5)
Time at 1–39 steps/min (min/day)	397.6 (92.4)	384.1 (92.9)	386.6 (92.2)	374.6 (96.6)	384.5 (94.3)	362.6 (102.6)	397.2 (91.3)
Time ≥40 steps/min (min/day)	24.8 (22.7)	21.7 (21.6)	22.6 (21.0)	18.2 (21.4)	22.6 (25.3)	20.6 (23.4)	24.2 (23.1)
Peak 30-min cadence	53.7 (25.9)	49.7 (25.1)	51.0 (24.9)	44.1 (23.9)	49.6 (24.8)	46.9 (28.6)	54.3 (28.1)

Abbreviations: min, minutes; MVPA, moderate to vigorous physical activity; VM, vector magnitude

Note: The means reported in the table are not adjusted.

**Table 3:** Self-reported walking pace by cancer type; the Women’s Health Accelerometry Collaboration.

	Cancer-Free n = 20,154	Any Cancer n = 2,554	Breast cancer n = 1,334	Colon cancer n = 175	Endometrial cancer n = 240	Lung cancer n = 93	Melanoma skin cancer n = 169
	n (%)	n (%)	n (%)	n (%)	n (%)	n (%)	n (%)
Does not walk regularly	2971 (14.7%)	424 (16.6%)	211 (15.8%)	27 (15.4%)	52 (21.7%)	23 (24.7%)	23 (13.6%)
Causal walking pace (<2 mph)	4246 (21.1%)	620 (24.3%)	337 (25.3%)	47 (26.9%)	49 (20.4%)	23 (24.7%)	34 (20.1%)
Normal walking pace (2–2.9 mph)	8131 (40.3%)	994 (38.9%)	508 (38.1%)	67 (38.3%)	98 (40.8%)	32 (34.4%)	79 (46.7%)
Brisk walking pace (3–3.9 mph)	4262 (21.1%)	447 (17.5%)	249 (18.7%)	25 (14.3%)	38 (15.8%)	13 (14.0%)	29 (17.2%)
Very brisk walking pace (> 4 mph)	315 (1.6%)	33 (1.3%)	14 (1.0%)	5 (2.9%)	3 (1.2%)	1 (1.1%)	3 (1.8%)
Missing	229 (1.1%)	36 (1.4%)	15 (1.1%)	4 (2.3%)	0 (0.0%)	1 (1.1%)	1 (0.6%)

Note: The percents reported in the table are not adjusted.

PA volume and intensity differed by type of cancer diagnosis (Table 2). Women with a history of colon cancer took on average 4,050 (SD = 2453) steps/day, spent 66.0 (SD = 41.4) minutes/day in MVPA, 18.2 (SD = 21.4) minutes/day in a stepping rate of ≥40 steps/minute, and had an average peak 30-minute cadence of 44.1 (SD = 23.9) steps/minute. Women with a history of melanoma skin cancer on average took 5,014 (SD = 2818) steps/day, spent 81.6 (SD = 47.0) minutes/day in MVPA, 24.2 (SD = 23.1) minutes/day in a stepping rate of ≥40 steps/minute, and had an average peak 30-minute cadence of 54.3 (SD = 28.1) steps/minute. Women with a history of lung cancer had slightly more average total steps but similar MVPA compared to than women with a history of colon cancer. Women with a history of breast or endometrial cancer had similar average total steps and MVPA; they were slightly less active than those with a history of melanoma. Reporting not walking regularly was higher among women with a history of lung cancer (24.7%) and endometrial cancer (21.7%) compared to other diagnoses (Table 3).

### Correlates of physical activity

**Fig 1:**
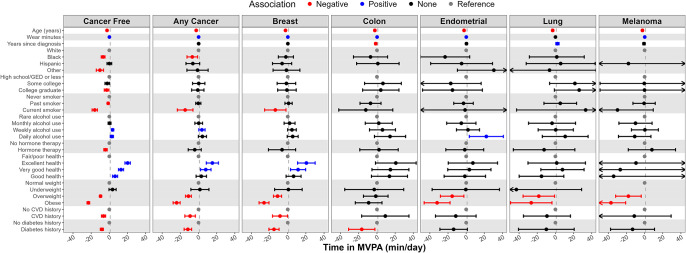
Correlates (independent variables) of time in moderate-to-vigorous physical activity (min/day, dependent variable) by cancer status and type; the Women’s Health Accelerometry Collaboration. Note: Any cancer does not include non-melanoma skin cancer. Models providing these estimates included age, race/ethnicity, education, smoking status, alcohol intake, menopausal hormone therapy use, general health, history of cardiovascular disease, history of diabetes, body mass index, accelerometer wear time, and years since cancer diagnosis (except in the cancer-free model). Each column represents a separate fully adjusted statistical model with estimates and 95% confidence intervals. Data for this figure can be found in S1 Table in S1 File.

**Fig 2:**
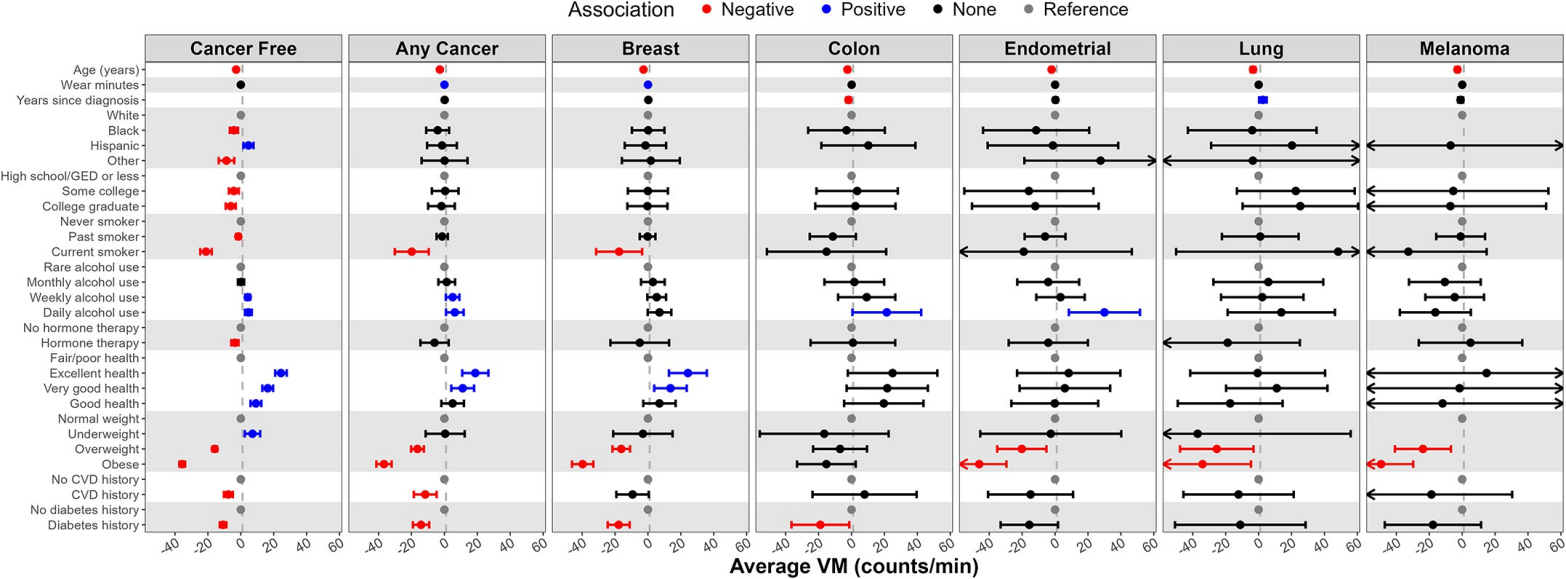
Correlates (independent variables) of average vector magnitude (VM, counts/min, dependent variable) by cancer status and type; the Women’s Health Accelerometry Collaboration. Note: Any cancer does not include non-melanoma skin cancer. Models providing these estimates included age, race/ethnicity, education, smoking status, alcohol intake, menopausal hormone therapy use, general health, history of cardiovascular disease, history of diabetes, body mass index, accelerometer wear time, and years since cancer diagnosis (except in the cancer-free model). Each column represents a separate fully adjusted statistical model with estimates and 95% confidence intervals. Data for this figure can be found in S2 Table in S1 File.

**Fig 3:**
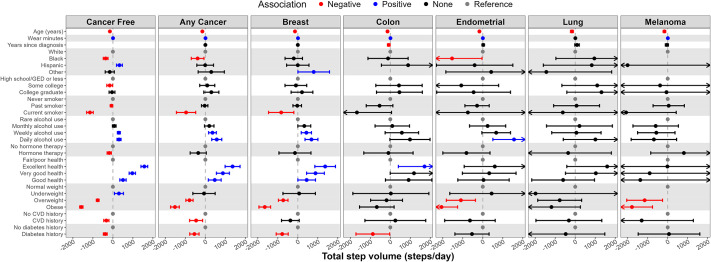
Correlates (independent variables) of total step volume (steps/day, dependent variable) by cancer status and type; the Women’s Health Accelerometry Collaboration. Note: Any cancer does not include non-melanoma skin cancer. Models providing these estimates included age, race/ethnicity, education, smoking status, alcohol intake, menopausal hormone therapy use, general health, history of cardiovascular disease, history of diabetes, body mass index, accelerometer wear time, and years since cancer diagnosis (except in the cancer-free model). Each column represents a separate fully adjusted statistical model with estimates and 95% confidence intervals. Data for this figure can be found in S3 Table in S1 File.

Figs 1–3 and S1, S2 Figs in S1 File present correlates of PA by cancer status (i.e., cancer free vs. any cancer) with numeric values found in S1-S5 Tables in S1 File. For those with and without cancer, age was inversely associated with all PA metrics. Black race was inversely associated with all PA metrics for cancer-free women and was inversely associated with MVPA and all step metrics for those with any cancer. Weekly and daily alcohol use was positively and current smoking was inversely associated with most PA metrics for women with and without cancer.

General health self-rated as excellent, very good, or good compared to fair/poor was positively associated with most PA metrics for women with and without cancer. Prior history of CVD, prior history of diabetes, overweight, and obesity were all inversely associated with most PA metrics for women with or without cancer. Current menopausal hormone therapy use was inversely associated with PA metrics for women without cancer. Among women with cancer, time since cancer diagnosis was not associated with the PA metrics.

Correlates of MVPA (Fig 1), average VM (Fig 2), total step volume (Fig 3), peak 30-minute cadence (S1 Fig in S1 File), and time at ≥40 steps/minute (S2 Fig in S1 File) are presented by cancer type (e.g., breast, colon, endometrial, lung, melanoma). Age was inversely associated with all PA metrics regardless of cancer type. Current smoking and diabetes history was inversely associated and excellent/very good self-rated health was positively associated with all PA metrics for women with breast cancer. Overweight and obesity were inversely associated with all PA metrics for women with breast, endometrial, and melanoma cancer. Higher education and hormone therapy had no association with PA metrics when examined by cancer type.

### Physical activity correlates: Stratified analysis

S6-S10 Tables in S1 File present correlates of PA by cancer status (i.e., cancer free vs. any cancer) and cohort (WHS vs. WHI/OPACH). Correlates of PA metrics were generally similar across the two cohorts, with a few differences described next. Among cancer-free WHI/OPACH women, Hispanic race/ethnicity was positively correlated for average VM/15-s and past smoking was inversely correlated to average VM/15-s, average total daily steps, and average peak 30-minute cadence. Among cancer-free WHS women, college graduation or higher education was positively correlated with all three step metrics while it was inversely correlated only for average total daily steps for cancer-free WHI/OPACH women.

Among WHS women with cancer, Black race/ethnicity was inversely correlated for MVPA. Among WHI/OPACH women with cancer, Hispanic race/ethnicity was positively correlated with average total daily steps.

### Sedentary behavior

Mean (SD) accelerometer-assessed time spent in SB was similar for those with (529.7 minutes/day, SD = 98.1) and without (521.7, SD = 101.2) cancer (Table 2). Mean SB was lowest for those with melanoma (522.9, SD = 94.9) and breast cancer (524.2, SD = 98.4), and highest for those with colon (549.7, SD = 97.3) and lung (544.5, SD = 108.5) cancers.

### Correlates of sedentary behavior

**Fig 4:**
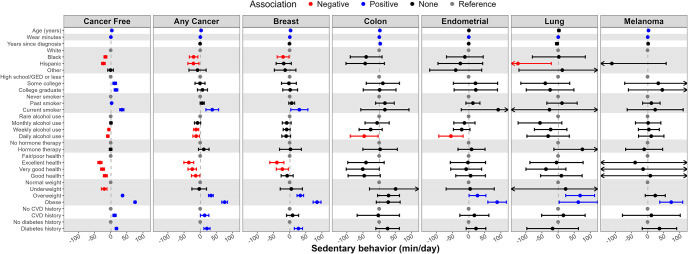
Correlates (independent variables) of sedentary behavior (min/day, dependent variable) by cancer status and type; the Women’s Health Accelerometry Collaboration. Note: Any cancer does not include non-melanoma skin cancer. Models providing these estimates included age, race/ethnicity, education, smoking status, alcohol intake, menopausal hormone therapy use, general health, history of cardiovascular disease, history of diabetes, body mass index, accelerometer wear time, and years since cancer diagnosis (except in the cancer-free model). Each column represents a separate fully adjusted statistical model with estimates and 95% confidence intervals. Data for this figure can be found in S4 Table in S1 File.

Fig 4 present correlates of SB by cancer status (i.e., cancer free vs. any cancer) with numeric values found in S11 Table in S1 File. For those with and without cancer, age was positively and Black and Hispanic race/ethnicity were inversely associated with SB. Among cancer-free women, having at least some college education was positively associated with SB. Among women with or without cancer, weekly or daily alcohol intake was inversely associated with SB, while current smoking was positively associated with SB. A history of CVD, a history of diabetes, overweight, and obesity were all positively associated with SB for women with and without cancer. A self-rated health of good, very good, or excellent was inversely associated with SB for women with and without cancer. For those with cancer, time since cancer diagnosis had no association with SB.

Correlates of SB (Fig 4) are presented by cancer type (e.g., breast, colon, endometrial, lung, melanoma). Hispanic ethnicity was inversely associated with SB for women with lung cancer. Current smoking was positively, and both very good and excellent general health were inversely associated with SB for women with breast cancer. Daily alcohol use was inversely associated with SB for women with colon and endometrial cancer. Overweight and obesity were positively associated with SB for women with breast, endometrial, and lung cancer; obesity was also positively associated with SB for women with melanoma skin cancer.

### Sedentary behavior correlates: Stratified analysis

S12 Table in S1 File presents correlates of sedentary behavior by cancer status (i.e., cancer free vs. any cancer) and cohort (WHS vs. WHI/OPACH). Correlates of SB were generally similar across the two cohorts, with a few differences identified among women with cancer. Specifically, among WHI/OPACH women with cancer, Black and Hispanic race/ethnicity and monthly or weekly alcohol intake were inversely correlated to SB.

## Discussion

The present study provides insight into PA and SB volume and their correlates in a population of postmenopausal women with (diagnosed an average of 8.7 years prior) and without a history of cancer. Levels of PA was generally higher for women without cancer, while SB was relatively similar for women with and without cancer. Average step volume and intensity were highest among women diagnosed with melanoma and lowest among women diagnosed with colon cancer. Age, smoking status, self-rated health, overweight, obesity, CVD, and diabetes generally had similar associations with PA and SB for women with and without cancer. However, by cancer type several correlates had null associations with PA and SB possibly due to small sample sizes for site-specific cancers.

Previous studies suggest a potential trend in higher levels of PA among cancer-free adults. For example, among 103,700 adults ages 40–69 years of age in the United Kingdom Biobank with cancer diagnoses less than 5 years prior to accelerometer data collection, cancer survivors engaged in less MVPA than cancer-free adults [34]. From the Baltimore Longitudinal Study of Aging, lower total PA was observed among cancer survivors compared to their cancer-free counterpart among 659 participants ages 50–69 years of age [9]. From the National Health and Nutrition Examination Survey (NHANES) 2003–2006 cohort, cancer survivors (n = 126) were also less likely to meet the PA guidelines compared to those with no prior cancer history [35].

In this study, older age, diabetes, overweight, and obesity were inversely associated with PA metrics regardless of cancer status. Aligning with our findings, Loprinzi et al. [36] found an inverse cross-sectional relationship between participation in MVPA and BMI among cancer survivors diagnosed with cancer more than 5 years ago in the NHANES 2003–2006 cohort. Further, age and BMI had an inverse relationship with PA in an examination of correlates of accelerometer measured PA among a cohort of breast cancer survivors ≥18 years of age in New Zealand [37]. Age was also found to have an inverse correlation with BMI and MVPA among colon cancer survivors from Alberta and Western Australia [38].

Our finding that SB was similar between those with and without cancer differs from NHANES results. Thraen-Borowski et al. [8] leveraged data from NHANES 2003–2006 cohort and examined light intensity PA and SB of cancer survivors. Cancer survivors engaged in less light PA and more SB than their non-cancer counterparts. However, the mean age was 12-years older for women in our present study (mean age 73.4 years) compared to the NHANES participants (mean age 61.4 years), which may partly account for the conflicting findings. Additionally, the present study leveraged vector magnitude (VM) from triaxial accelerometers to define SB whereas, Thraen-Borowski et al. [8] utilized a less precise measure of counts per minute from uniaxial accelerometers.

Prior research examining correlates of SB partially align with our current findings. The present study found that age, overweight, obesity, current smoking, CVD, and diabetes were positively associated with SB, whereas Hispanic, Black, weekly/daily alcohol intake, and excellent/very good/good self-rated health were inversely associated with SB for both cancer and cancer-free women. Consistent with our findings, in another study examination of lung cancer survivors (mean age = 71 years) found SB was positively associated with BMI; however, in contrast to our findings among lung cancer survivors, positive associations with age and smoking were reported [17]. Smoking status definitions differed between our study and this prior study and may account for the conflicting findings. Further, due to sample size, correlates among lung cancer survivors in the present study should be interpreted with caution.

### Strengths and limitations

There were several notable strengths of the present study. Pooling data from WHS and WHI/OPACH afforded the opportunity to explore correlates of PA and SB in several cancer types over a relatively wide age range. Use of accelerometry provides robust estimates of a spectrum of PA and SB and limits potential misclassification. The prospective nature and long follow-up period for the cohorts enabled ascertainment of multiple cancer diagnoses and allowed for assessment of years since diagnosis as a correlate.

Our findings should also be considered in light of several limitations. The thresholds used to define PA intensity (e.g., light, MVPA) were derived from a laboratory-based study and have not been verified in free-living individuals [32]. While the reported amount of MVPA may seem high, this lifestyle measure falls within the range of 10.8 to 106.8 minutes/day reported from a representative sample of United States adults 60 years and older that considered a range of accelerometry cutpoints [39]. Examination of differences in SB and PA by cancer type was limited, since our sample size, and thus statistical power, for site-specific cancers was limited. This may have also contributed to some differences by cohort. Confidence intervals for strata such as race/ethnicity were wider in sparser cancers and should be interpreted with caution. We were bound by correlates collected similarly across the cohorts. Correlates assessed in prior studies such as marital status, income, occupation, blood pressure, and depression were unable to be assessed [19, 40] as the measures were not harmonized across cohorts. We also could not examine how cancer-related outcomes and side-effects of treatments impacted engagement in PA years after cancer. Considering the socio-ecologic model [41], we did not capture potential interpersonal, community, policy, and environmental correlates, which may provide additional context around barriers and facilitators for engaging in PA. Additionally, due to the cross-sectional nature of the study, causal relationships cannot be drawn and reverse causation of associations may exist.

Further, in the present study, findings may reflect associations captured that have been mediated through other correlates rather than the singular correlate itself. Research identifying mediators versus confounders should be considered for future research in this field. Cancer survivors may have lower levels of PA than cancer-free adults because low-levels of PA may have contributed to cancer development; alternatively, the effects of the cancer and/or treatment may have limited PA levels. Further, higher levels of PA among melanoma patients compared to other cancers may be due to sun exposure during PA. Age may also play a role in the type of cancer present as well as PA capability; melanoma occurs in a younger patient population and consequently may consist of a more physically capable population. Selection bias may be present as participants were required to be able to walk unassisted to participate, which was a criterion for the type of accelerometer at the initiation of the two studies. Cancer burden and treatment and cancer may be associated with limited mobility. Therefore, levels of PA may systematically differ by severity of cancer and treatment. Further, patients with limited mobility due to disease progression and treatment may be less likely to enroll in a study, thereby, there may be an underestimation of cancer survivors within the population. Findings from this study thereby are limited in generalizability to a population of relatively mobile postmenopausal women. Finally, this study was comprised solely of volunteer women, thus findings reported here may not be generalizable to other populations such as men.

## Conclusion

In a cohort of postmenopausal women, cancer survivors had slightly lower average PA volumes and intensities than cancer-free participants, whereas SB was relatively similar for women with and without cancer. While PA volume and intensity differed by type of cancer diagnosis, SB did not meaningfully vary across type of cancer diagnosis. Several sociodemographic, health, and chronic conditions were correlates of PA/SB for postmenopausal women with and without cancer. Further research is needed with adequate sample sizes to examine differences in PA and SB by cancer type, ideally longitudinal in design to gain insight into potential determinants of PA/SB. Understanding patterns, correlates, and determinants of PA and SB among cancer survivors may support development of targeted interventions promoting behavioral changes to improve PA levels among cancer survivors.

## Supporting information

S1 File (PDF)
