# Supplementary material for: Correlates of physical activity and sedentary behavior among cancer survivors and cancer-free women: The Women’s Health Accelerometry Collaboration
Source: PLoS One. 2024 Apr 4;19(4):e0301233. doi: 10.1371/journal.pone.0301233 (PMC10994363; doi:10.1371/journal.pone.0301233)
Supplement: S1 File — (PDF) [file pone.0301233.s001.pdf]

Supplemental Content

Supplemental Figure 1. Correlates (independent variables) of Peak 30-minute Cadence (steps/min, dependent variable) by Cancer Status and Type; the Women's Health Accelerometry Collaboration

Supplemental Figure 2. Correlates (independent variables) of Time (min/day) at $\geq$40 steps/minute (dependent variable) by Cancer Status and Type; the Women's Health Accelerometry Collaboration

Supplemental Table 1. Data from Figure 1: Correlates (independent variables) of Time in Moderate-to-Vigorous Physical Activity (min/day, dependent variable) by Cancer Status and Type; the Women's Health Accelerometry Collaboration

Supplemental Table 2. Data from Figure 2: Correlates (independent variables) of Average Vector Magnitude (VM, counts/min, dependent variable) by Cancer Status and Type; the Women's Health Accelerometry Collaboration

Supplemental Table 3. Data from Figure 3: Correlates (independent variables) of Total Step Volume (steps/day, dependent variable) by Cancer Status and Type; the Women's Health Accelerometry Collaboration

Supplemental Table 4. Data from Supplemental Figure 1: Correlates (independent variables) of Peak 30-minute Cadence (steps/min, dependent variable) by Cancer Status and Type; the Women's Health Accelerometry Collaboration

Supplemental Table 5. Data from Supplemental Figure 2: Correlates (independent variables) of Time (min/day, dependent variable) at $\geq$40 steps/minute by Cancer Status and Type; the Women's Health Accelerometry Collaboration

Supplemental Table 6: Correlates (independent variables) of Time in Moderate-to-Vigorous Physical Activity (min/day, dependent variable) by Cancer Status and Cohort; the Women's Health Accelerometry Collaboration

Supplemental Table 7: Correlates (independent variables) of Average Vector Magnitude (VM, counts/min, dependent variable) by Cancer Status and Cohort; the Women's Health Accelerometry Collaboration

Supplemental Table 8: Correlates (independent variables) of Total Step Volume (steps/day, dependent variable) by Cancer Status and Cohort; the Women's Health Accelerometry Collaboration

Supplemental Table 9: Correlates (independent variables) of Peak 30-minute Cadence (steps/min, dependent variable) by Cancer Status and Cohort; the Women's Health Accelerometry Collaboration

Supplemental Table 10: Correlates (independent variables) of Time (min/day, dependent variable) at $\geq$40 steps/minute by Cancer Status and Cohort; the Women's Health Accelerometry Collaboration

Supplemental Table 11. Data from Figure 4: Correlates (independent variables) of Sedentary Behavior (min/day, dependent variable) by Cancer Status and Type; the Women's Health Accelerometry Collaboration

Supplemental Table 12: Correlates (independent variables) of Sedentary Behavior (min/day, dependent variable) by Cancer Status and Cohort; the Women’s Health Accelerometry Collaboration

Supplemental Figure 1. Correlates (independent variables) of Peak 30-minute Cadence (steps/min, dependent variable) by Cancer Status and Type; the Women's Health Accelerometry Collaboration

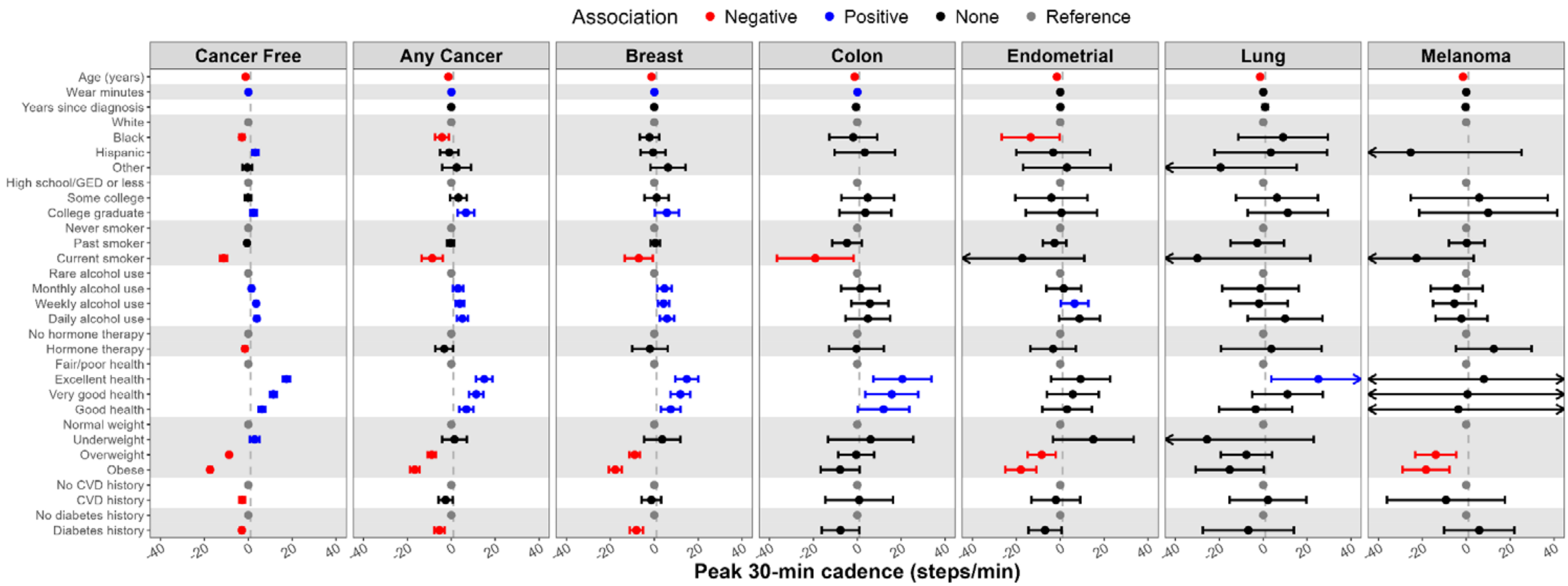

Abbreviations: CVD, cardiovascular disease; GED, general educational diploma

Note: Any cancer does not include non-melanoma skin cancer. Models providing these estimates included age, race/ethnicity, education, smoking status, alcohol intake, menopausal hormone therapy use, general health, history of cardiovascular disease, history of diabetes, body mass index, accelerometer wear time, and years since cancer diagnosis (except in the cancer-free model). Each column represents a separate fully adjusted statistical model with estimates and 95% confidence intervals. Data for this figure can be found in Supplemental Table 5.

Supplemental Figure 2. Correlates (independent variables) of Time (min/day) at ≥40 steps/minute (dependent variable) by Cancer Status and Type; the Women's Health Accelerometry Collaboration

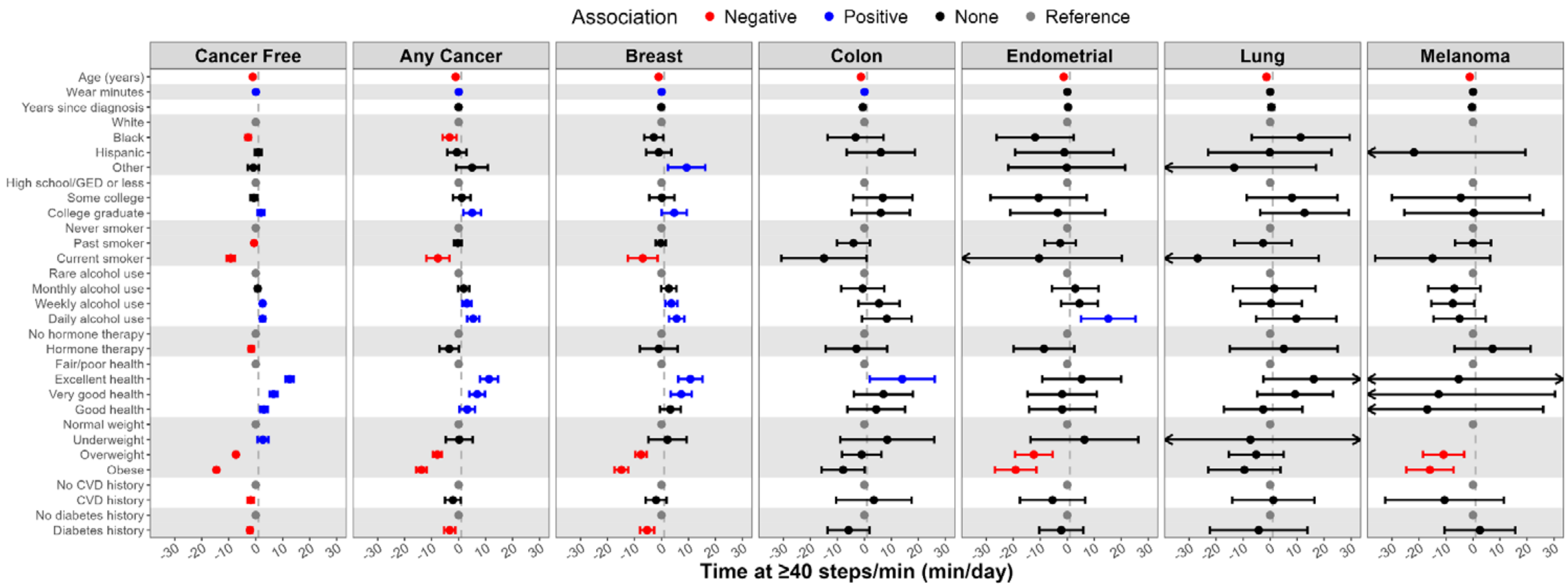

Abbreviations: CVD, cardiovascular disease; GED, general educational diploma

Note: Any cancer does not include non-melanoma skin cancer. Models providing these estimates included age, race/ethnicity, education, smoking status, alcohol intake, menopausal hormone therapy use, general health, history of cardiovascular disease, history of diabetes, body mass index, accelerometer wear time, and years since cancer diagnosis (except in the cancer-free model). Each column represents a separate fully adjusted statistical model estimates with estimates and 95% confidence intervals. Data for this figure can be found in Supplemental Table 6.

Supplemental Table 1. Data from Figure 1: Correlates (independent variables) of Time in Moderate-to-Vigorous Physical Activity (min/day, dependent variable) by Cancer Status and Type; the Women's Health Accelerometry Collaboration

Characteristic	*Level*	*Cancer-free*	*Any cancer*	*Breast cancer*	*Colon cancer*	*Endometrial cancer*	*Lung cancer*	*Melanoma skin cancer*
Age		-2.5 (-2.6, -2.4)	-2.5 (-2.8, -2.3)	-2.5 (-2.8, -2.1)	-2.3 (-3.2, -1.5)	-2.3 (-3.1, -1.5)	-3.0 (-4.1, -1.9)	-2.4 (-3.4, -1.4)
Race/ethnicity								
	Black	-6.8 (-8.9, -4.7)	-6.9 (-12.7, -1.0)	-2.0 (-10.4, 6.3)	-7.1 (-25.9, 11.7)	-23.3 (-50.4, 3.9)	1.6 (-28.7, 31.8)	NA
	Hispanic	0.1 (-2.5, 2.6)	-6.3 (-13.8, 1.3)	-6.2 (-16.8, 4.4)	1.0 (-22.4, 24.4)	-5.1 (-39.1, 29.0)	5.8 (-32.2, 43.8)	-17.8 (-94.2, 58.5)
	Other	-10.4 (-14.4, -6.3)	-1.1 (-12.8, 10.6)	-1.4 (-16.2, 13.4)	NA	30.5 (-9.7, 70.7)	-6.5 (-57.7, 44.8)	NA
	White	Ref	Ref	Ref	Ref	Ref	Ref	Ref
Education level								
	Some College	-2.2 (-4.7, 0.4)	0.2 (-6.5, 7.0)	-1.5 (-11.7, 8.8)	6.6 (-13.7, 26.8)	-17.0 (-50.5, 16.5)	21.5 (-6.4, 49.3)	-0.4 (-49.1, 48.3)
	College Graduate	-3.0 (-5.5, -0.4)	-0.8 (-7.6, 6.0)	-0.9 (-11.1, 9.4)	4.2 (-15.8, 24.2)	-15.1 (-48.0, 17.9)	26.2 (-1.1, 53.5)	-0.1 (-49.1, 48.9)
	High School/GED or less	Ref	Ref	Ref	Ref	Ref	Ref	Ref
Smoking								
	Past	-1.2 (-2.3, -0.1)	-0.2 (-3.1, 2.6)	0.9 (-3.1, 4.9)	-7.1 (-18.7, 4.5)	-2.9 (-13.7, 7.8)	5.4 (-12.6, 23.4)	-0.6 (-12.9, 11.7)
	Current	-16.0 (-19.0, -13.1)	-14.7 (-23.4, -6.1)	-13.7 (-25.5, -1.9)	-12.3 (-42.1, 17.5)	-1.2 (-58.2, 55.8)	33.3 (-42.8, 109.4)	-29.9 (-69.3, 9.5)
	Never	Ref	Ref	Ref	Ref	Ref	Ref	Ref
Alcohol intake								
	Monthly	0.3 (-1.3, 1.9)	0.2 (-4.0, 4.5)	2.0 (-4.0, 8.0)	2.0 (-12.9, 16.9)	-5.3 (-21.5, 10.8)	-3.7 (-29.6, 22.2)	-10.0 (-28.3, 8.2)
	Weekly	3.6 (2.3, 4.9)	3.8 (0.3, 7.2)	4.6 (-0.1, 9.4)	6.1 (-8.2, 20.4)	2.0 (-10.7, 14.7)	0.4 (-19.0, 19.8)	0.3 (-14.5, 15.2)
	Daily	2.8 (1.0, 4.5)	4.3 (-0.2, 8.8)	5.4 (-0.6, 11.5)	14.4 (-2.8, 31.6)	22.2 (3.4, 40.9)	10.8 (-14.4, 36.0)	-10.9 (-28.7, 7.0)
	Never or rarely	Ref	Ref	Ref	Ref	Ref	Ref	Ref
Current hormone therapy use								
	Yes	-4.2 (-6.1, -2.3)	-4.2 (-11.4, 3.0)	-6.3 (-21.3, 8.7)	2.2 (-18.9, 23.4)	-1.5 (-22.4, 19.3)	-12.4 (-46.3, 21.5)	8.0 (-18.0, 34.1)
	No	Ref	Ref	Ref	Ref	Ref	Ref	Ref
Self-rated health								
	Excellent	20.2 (17.2, 23.2)	15.2 (8.6, 21.9)	20.5 (10.9, 30.2)	20.8 (-1.6, 43.2)	7.4 (-19.7, 34.6)	1.5 (-30.2, 33.1)	-9.5 (-90.7, 71.7)
	Very Good	12.9 (10.1, 15.6)	8.0 (2.2, 13.8)	11.4 (3.1, 19.7)	14.8 (-5.5, 35.2)	3.5 (-20.3, 27.3)	7.8 (-16.0, 31.6)	-26.8 (-107.0, 53.4)
	Good	6.4 (3.6, 9.1)	3.0 (-2.7, 8.8)	6.4 (-1.8, 14.7)	13.8 (-6.1, 33.6)	-1.5 (-24.3, 21.3)	-15.3 (-39.9, 9.3)	-34.1 (-114.0, 45.8)
	Fair/poor	Ref	Ref	Ref	Ref	Ref	Ref	Ref
Body mass index								
	Underweight	3.6 (-0.4, 7.5)	1.5 (-8.4, 11.5)	0.7 (-14.5, 15.9)	-3.0 (-35.5, 29.5)	-0.4 (-37.5, 36.7)	-43.0 (-114.9, 28.9)	NA
	Overweight	-9.7 (-10.9, -8.4)	-11.1 (-14.3, -7.9)	-11.2 (-15.7, -6.7)	-1.3 (-14.9, 12.2)	-15.4 (-28.4, -2.5)	-18.3 (-35.6, -0.9)	-17.8 (-31.9, -3.7)
	Obese	-23.1 (-24.5, -21.6)	-24.1 (-28.0, -20.2)	-26.1 (-31.6, -20.7)	-9.2 (-23.9, 5.6)	-32.0 (-46.3, -17.7)	-26.6 (-49.4, -3.8)	-37.0 (-53.2, -20.9)
	Normal weight	Ref	Ref	Ref	Ref	Ref	Ref	Ref
History of cardiovascular disease								
	Yes	-6.3 (-8.6, -4.1)	-9.3 (-15.1, -3.5)	-8.5 (-16.8, -0.2)	9.2 (-16.9, 35.2)	-11.5 (-33.8, 10.9)	-9.3 (-35.2, 16.6)	-11.4 (-52.0, 29.2)
	No	Ref	Ref	Ref	Ref	Ref	Ref	Ref
History of diabetes								
	Yes	-8.2 (-9.9, -6.5)	-11.7 (-15.9, -7.6)	-15.1 (-20.8, -9.5)	-16.8 (-31.2, -2.3)	-13.8 (-28.9, 1.3)	-10.0 (-40.7, 20.7)	-13.3 (-37.5, 10.9)
	No	Ref	Ref	Ref	Ref	Ref	Ref	Ref
Accelerometer wear time		0.1 (0.1, 0.1)	0.1 (0.1, 0.1)	0.1 (0.1, 0.1)	0.1 (0.1, 0.2)	0.1 (0.0, 0.2)	0.1 (-0.1, 0.2)	0.1 (0.0, 0.2)
Time since cancer diagnosis		NA	0.1 (-0.2, 0.4)	0.1 (-0.3, 0.5)	-1.3 (-2.5, -0.2)	0.3 (-0.7, 1.3)	2.3 (0.5, 4.0)	-0.7 (-1.9, 0.5)

Abbreviations: GED, general educational diploma; NA, not applicable; Ref, referent

Note: Any cancer does not include non-melanoma skin cancer. Models providing these estimates included age, race/ethnicity, education, smoking status, alcohol intake, menopausal hormone therapy use, general health, history of cardiovascular disease, history

of diabetes, body mass index, accelerometer wear time, and years since cancer diagnosis (except in the cancer-free model). Each column represents a separate fully adjusted statistical model with estimates and 95% confidence intervals.

Supplemental Table 2. Data from Figure 2: Correlates (independent variables) of Average Vector Magnitude (VM, counts/min, dependent variable) by Cancer Status and Type; the Women's Health Accelerometry Collaboration

Characteristic	*Level*	*Cancer-free*	*Any cancer*	*Breast cancer*	*Colon cancer*	*Endometrial cancer*	*Lung cancer*	*Melanoma skin cancer*
Age		-2.8 (-3.0, -2.7)	-2.8 (-3.0, -2.5)	-2.7 (-3.1, -2.3)	-2.4 (-3.5, -1.4)	-2.1 (-3.1, -1.1)	-3.4 (-4.8, -1.9)	-2.9 (-4.1, -1.7)
Race/ethnicity								
	Black	-4.2 (-6.7, -1.7)	-4.1 (-11.1, 2.9)	0.1 (-9.7, 10.0)	-3.0 (-26.4, 20.3)	-11.5 (-43.8, 20.8)	-3.9 (-43.0, 35.2)	NA
	Hispanic	4.7 (1.6, 7.7)	-1.4 (-10.5, 7.6)	-1.5 (-14.1, 11.1)	10.3 (-18.3, 38.9)	-1.3 (-41.0, 38.4)	20.3 (-28.9, 69.5)	-7.0 (-99.3, 85.2)
	Other	-8.7 (-13.5, -4.0)	0.1 (-13.8, 14.1)	1.8 (-15.8, 19.4)	NA	27.7 (-18.7, 74.0)	-3.5 (-69.9, 62.8)	NA
	White	Ref	Ref	Ref	Ref	Ref	Ref	Ref
Education level								
	Some College	-4.2 (-7.3, -1.2)	0.5 (-7.6, 8.6)	-0.04 (-12.21, 12.14	3.4 (-21.3, 28.1)	-15.9 (-55.1, 23.3)	22.6 (-13.2, 58.4)	-5.4 (-63.2, 52.4)
	College Graduate	-6.1 (-9.2, -3.1)	-1.8 (-9.9, 6.3)	-0.3 (-12.5, 11.9)	2.4 (-22.0, 26.7)	-12.0 (-50.5, 26.5)	25.3 (-9.8, 60.4)	-7.2 (-65.4, 51.0)
	High School/GED or less	Ref	Ref	Ref	Ref	Ref	Ref	Ref
Smoking								
	Past	-1.5 (-2.8, -0.3)	-1.3 (-4.7, 2.1)	-0.2 (-5.0, 4.6)	-11.3 (-25.4, 2.7)	-6.1 (-18.5, 6.4)	1.0 (-22.3, 24.3)	-0.9 (-15.8, 13.9)
	Current	-21.1 (-24.6, -17.7)	-19.8 (-30.2, -9.5)	-17.5 (-31.5, -3.5)	-15.2 (-51.4, 21.1)	-19.0 (-84.8, 46.7)	48.3 (-50.1, 146.8)	-32.7 (-80.3, 14.9)
	Never	Ref	Ref	Ref	Ref	Ref	Ref	Ref
Alcohol intake								
	Monthly	0.1 (-1.8, 2.0)	1.4 (-3.6, 6.5)	3.0 (-4.2, 10.2)	1.7 (-16.4, 19.9)	-4.2 (-23.0, 14.7)	5.9 (-27.5, 39.3)	-10.5 (-32.3, 11.3)
	Weekly	4.2 (2.7, 5.7)	5.0 (0.9, 9.1)	5.3 (-0.4, 11.0)	9.2 (-8.2, 26.6)	3.3 (-11.4, 18.0)	2.2 (-22.8, 27.2)	-4.5 (-22.4, 13.3)
	Daily	4.6 (2.5, 6.7)	6.4 (1.0, 11.7)	7.0 (-0.1, 14.2)	21.5 (0.6, 42.3)	30.0 (8.4, 51.6)	13.8 (-18.8, 46.4)	-16.3 (-37.9, 5.3)
	Never or rarely	Ref	Ref	Ref	Ref	Ref	Ref	Ref
Current hormone therapy use								
	Yes	-3.6 (-5.8, -1.4)	-6.0 (-14.6, 2.6)	-5.0 (-22.8, 12.9)	0.9 (-24.9, 26.6)	-4.1 (-28.2, 20.0)	-18.8 (-62.7, 25.1)	5.1 (-26.4, 36.5)
	No	Ref	Ref	Ref	Ref	Ref	Ref	Ref
Self-rated health								
	Excellent	24.4 (20.9, 27.9)	18.8 (10.8, 26.8)	24.3 (12.8, 35.8)	25.0 (-2.2, 52.2)	8.3 (-23.1, 39.7)	-0.6 (-41.6, 40.4)	14.9 (-83.0, 112.7)
	Very Good	16.3 (13.1, 19.6)	11.1 (4.2, 18.0)	13.7 (3.8, 23.6)	21.7 (-3.0, 46.4)	6.0 (-21.6, 33.5)	11.0 (-19.8, 41.8)	-1.6 (-98.1, 95.0)
	Good	9.2 (5.9, 12.5)	5.0 (-1.8, 11.9)	7.0 (-2.8, 16.8)	19.6 (-4.5, 43.7)	-0.2 (-26.6, 26.2)	-17.3 (-49.1, 14.6)	-11.9 (-108.1, 84.3)
	Fair/poor	Ref	Ref	Ref	Ref	Ref	Ref	Ref
Body mass index								
	Underweight	7.1 (2.4, 11.8)	0.5 (-11.4, 12.4)	-3.0 (-21.1, 15.0)	-16.6 (-55.7, 22.6)	-2.6 (-45.4, 40.3)	-37.1 (-130.1, 56.0)	NA
	Overweight	-15.9 (-17.3, -14.4)	-16.3 (-20.2, -12.5)	-16.2 (-21.6, -10.9)	-7.0 (-23.3, 9.4)	-20.2 (-35.2, -5.2)	-25.4 (-47.7, -3.1)	-23.8 (-40.8, -6.8)
	Obese	-35.6 (-37.3, -33.9)	-36.7 (-41.3, -32.1)	-39.7 (-46.2, -33.2)	-15.3 (-33.1, 2.6)	-46.1 (-62.6, -29.5)	-34.1 (-63.5, -4.6)	-49.3 (-68.8, -29.7)
	Normal weight	Ref	Ref	Ref	Ref	Ref	Ref	Ref
History of cardiovascular disease								
	Yes	-7.6 (-10.3, -4.9)	-11.6 (-18.5, -4.7)	-9.3 (-19.2, 0.5)	7.9 (-23.7, 39.6)	-14.9 (-40.7, 11.0)	-12.2 (-45.7, 21.3)	-18.7 (-67.7, 30.4)
	No	Ref	Ref	Ref	Ref	Ref	Ref	Ref
History of diabetes								
	Yes	-10.8 (-12.8, -8.8)	-14.2 (-19.1, -9.2)	-17.8 (-24.5, -11.1)	-18.9 (-36.5, -1.3)	-15.6 (-33.1, 1.8)	-11.1 (-50.8, 28.6)	-17.8 (-47.0, 11.5)
	No	Ref	Ref	Ref	Ref	Ref	Ref	Ref
Accelerometer wear time		0.001 (-0.007, 0.009	0.033 (0.011, 0.056	0.034 (0.001, 0.067	0.07 (-0.03, 0.17)	0.04 (-0.04, 0.11)	0.02 (-0.13, 0.17)	0.04 (-0.06, 0.13)
Time since cancer diagnosis		NA	0.2 (-0.2, 0.5)	0.2 (-0.2, 0.7)	-1.8 (-3.2, -0.5)	0.3 (-0.9, 1.4)	2.6 (0.3, 4.9)	-1.0 (-2.5, 0.5)

Abbreviations: GED, general educational diploma; NA, not applicable; Ref, referent

Note: Any cancer does not include non-melanoma skin cancer. Models providing these estimates included age, race/ethnicity, education, smoking status, alcohol intake, menopausal hormone therapy use, general health, history of cardiovascular disease, history of diabetes, body mass index,

accelerometer wear time, and years since cancer diagnosis (except in the cancer-free model). Each column represents a separate fully adjusted statistical model with estimates and 95% confidence intervals.

Supplemental Table 3. Data from Figure 3: Correlates (independent variables) of Total Step Volume (steps/day, dependent variable) by Cancer Status and Type; the Women's Health Accelerometry Collaboration

Characteristic	*Level*	*Cancer-free*	*Any cancer*	*Breast cancer*	*Colon cancer*	*Endometrial cancer*	*Lung cancer*	*Melanoma skin cancer*
Age		-150.1 (-155.4, -144.8)	-149.9 (-163.2, -136.6)	-142.6 (-161.0, -124.3)	-138.7 (-185.0, -92.4)	-167.1 (-211.4, -122.8)	-164.4 (-235.9, -92.9)	-163.8 (-225.4, -102.2)
Race/ethnicity								
	Black	-363.6 (-482.2, -245.0)	-387.6 (-705.3, -69.8)	-202.9 (-639.3, 233.4)	-113.2 (-1117.9, 891.5)	-1535.9 (-3035.4, -36.4)	956.2 (-968.4, 2880.7)	NA
	Hispanic	327.3 (180.3, 474.2)	-9.8 (-427.6, 408.0)	-1.6 (-561.2, 558.1)	891.0 (-445.5, 2227.4)	-407.3 (-2319.1, 1504.5)	825.6 (-1594.8, 3245.9)	-2005.8 (-6864.6, 2853.0)
	Other	-157.6 (-385.2, 70.0)	294.1 (-361.4, 949.5)	794.5 (4.0, 1585.1)		420.7 (-1847.1, 2688.5)	-1446.6 (-4715.7, 1822.4)	NA
	White	Ref	Ref	Ref	Ref	Ref	Ref	Ref
Education level								
	Some College	-158.9 (-304.1, -13.6)	94.5 (-282.1, 471.1)	-98.5 (-642.1, 445.2)	434.3 (-725.1, 1593.7)	-1075.3 (-2943.7, 793.1)	1099.3 (-668.4, 2866.9)	-381.6 (-3384.1, 2621.0)
	College Graduate	-44.4 (-190.4, 101.5)	298.6 (-78.4, 675.6)	207.3 (-336.4, 751.1)	456.6 (-690.4, 1603.7)	-458.3 (-2302.6, 1386.0)	1311.5 (-420.6, 3043.6)	-62.4 (-3088.6, 2963.8)
	High School/GED or less	Ref	Ref	Ref	Ref	Ref	Ref	Ref
Smoking								
	Past	-70.8 (-132.8, -8.9)	-34.2 (-194.4, 126.1)	-29.7 (-243.9, 184.5)	-537.0 (-1203.5, 129.5)	-285.3 (-893.5, 322.8)	66.3 (-1081.5, 1214.2)	49.7 (-732.7, 832.1)
	Current	-1148.5 (-1314.7, -982.3)	-964.6 (-1446.9, -482.3)	-832.2 (-1464.5, -199.9)	-1668.9 (-3399.6, 61.7)	-745.6 (-3965.2, 2474.1)	-622.1 (-5474.2, 4230.0)	-2080.7 (-4587.4, 426.0)
	Never	Ref	Ref	Ref	Ref	Ref	Ref	Ref
Alcohol intake								
	Monthly	68.0 (-23.1, 159.1)	195.1 (-40.8, 430.9)	322.9 (-0.1, 645.8)	99.0 (-761.9, 960.0)	241.3 (-663.1, 1145.6)	211.6 (-1434.8, 1858.0)	-601.2 (-1737.9, 535.6)
	Weekly	298.3 (225.2, 371.5)	356.0 (163.1, 548.9)	426.5 (170.8, 682.3)	577.6 (-258.4, 1413.5)	670.7 (-45.1, 1386.4)	57.7 (-1175.9, 1291.3)	-570.2 (-1505.3, 365.0)
	Daily	307.5 (207.8, 407.2)	569.6 (318.4, 820.9)	681.0 (358.6, 1003.4)	983.2 (-20.1, 1986.6)	1564.3 (504.9, 2623.6)	1008.1 (-598.5, 2614.7)	-691.0 (-1827.6, 445.7)
	Never or rarely	Ref	Ref	Ref	Ref	Ref	Ref	Ref
Current hormone therapy use								
	Yes	-181.8 (-288.6, -75.0)	-356.5 (-760.1, 47.0)	-143.8 (-945.0, 657.4)	-93.1 (-1325.8, 1139.5)	-817.5 (-1996.9, 361.9)	-355.1 (-2516.9, 1806.8)	808.8 (-849.0, 2466.5)
	No	Ref	Ref	Ref	Ref	Ref	Ref	Ref
Self-rated health								
	Excellent	1572.0 (1403.8, 1740.3)	1360.6 (986.8, 1734.5)	1366.4 (849.9, 1882.8)	1711.5 (403.6, 3019.4)	600.7 (-930.3, 2131.6)	1597.1 (-421.8, 3616.0)	-14.0 (-5161.6, 5133.6)
	Very Good	970.0 (812.5, 1127.5)	876.4 (552.1, 1200.7)	882.6 (438.9, 1326.4)	1183.8 (-4.7, 2372.2)	322.7 (-1019.2, 1664.6)	1029.6 (-487.7, 2547.0)	-901.6 (-5976.8, 4173.5)
	Good	503.9 (346.1, 661.7)	470.2 (148.7, 791.7)	441.9 (0.3, 883.5)	916.6 (-242.6, 2075.8)	36.3 (-1248.4, 1321.0)	-606.0 (-2175.6, 963.6)	-1371.3 (-6425.9, 3683.3)
	Fair/poor	Ref	Ref	Ref	Ref	Ref	Ref	Ref
Body mass index								
	Underweight	301.7 (76.5, 527.0)	-67.9 (-626.3, 490.4)	66.7 (-744.8, 878.1)	28.3 (-1872.7, 1929.3)	442.5 (-1649.5, 2534.6)	-1985.4 (-6569.4, 2598.6)	NA
	Overweight	-759.7 (-829.0, -690.3)	-794.2 (-976.2, -612.1)	-731.0 (-970.9, -491.1)	-187.2 (-984.8, 610.4)	-1093.9 (-1823.3, -364.5)	-777.6 (-1875.7, 320.5)	-1153.0 (-2049.5, -256.6)
	Obese	-1578.4 (-1661.4, -1495.4)	-1521.0 (-1739.1, -1303.0)	-1652.9 (-1944.9, -1360.9)	-674.3 (-1538.9, 190.3)	-2062.6 (-2866.1, -1259.1)	-1189.4 (-2636.9, 258.1)	-1787.6 (-2816.5, -758.7)
	Normal weight	Ref	Ref	Ref	Ref	Ref	Ref	Ref
History of cardiovascular disease								
	Yes	-326.3 (-455.9, -196.6)	-462.6 (-787.1, -138.1)	-372.9 (-815.0, 69.2)	251.4 (-1269.2, 1771.9)	-641.1 (-1904.6, 622.3)	-318.7 (-1966.0, 1328.5)	-1302.5 (-3887.7, 1282.6)
	No	Ref	Ref	Ref	Ref	Ref	Ref	Ref
History of diabetes								
	Yes	-381.4 (-477.9, -284.9)	-548.6 (-781.7, -315.6)	-782.0 (-1082.6, -481.4)	-873.9 (-1718.0, -29.8)	-539.3 (-1394.1, 315.6)	-475.8 (-2431.1, 1479.5)	64.4 (-1477.2, 1606.0)
	No	Ref	Ref	Ref	Ref	Ref	Ref	Ref
Accelerometer wear time		6.0 (5.6, 6.4)	6.7 (5.6, 7.8)	7.4 (5.9, 8.8)	7.6 (2.8, 12.4)	5.1 (1.3, 8.8)	5.6 (-1.9, 13.1)	6.1 (1.1, 11.1)
Time since cancer diagnosis		NA	0.8 (-14.8, 16.3)	-0.8 (-21.3, 19.8)	-75.6 (-141.6, -9.6)	22.8 (-34.5, 80.1)	89.5 (-23.1, 202.1)	-41.0 (-118.6, 36.5)

Abbreviations: GED, general educational diploma; NA, not applicable; Ref, referent

Note: Any cancer does not include non-melanoma skin cancer. Models providing these estimates included age, race/ethnicity, education, smoking status, alcohol intake, menopausal hormone therapy use, general health, history of cardiovascular disease, history of diabetes, body mass index, accelerometer wear time, and years since cancer diagnosis (except in the cancer-free model). Each column represents a separate fully adjusted statistical model with estimates and 95% confidence intervals.

Supplemental Table 4. Data from Supplemental Figure 1: Correlates (independent variables) of Peak 30-minute Cadence (steps/min, dependent variable) by Cancer Status and Type; the Women's Health Accelerometry Collaboration

Characteristic	*Level*	*Cancer-free*	*Any cancer*	*Breast cancer*	*Colon cancer*	*Endometrial cancer*	*Lung cancer*	*Melanoma skin cancer*
Age		-1.2 (-1.3, -1.2)	-1.3 (-1.4, -1.2)	-1.2 (-1.4, -1.1)	-1.2 (-1.7, -0.7)	-1.5 (-1.9, -1.1)	-1.4 (-2.2, -0.6)	-1.5 (-2.1, -0.8)
Race/ethnicity								
	Black	-2.9 (-4.1, -1.7)	-4.2 (-7.4, -1.1)	-2.2 (-6.6, 2.3)	-1.8 (-12.7, 9.1)	-13.4 (-26.7, -0.2)	9.1 (-11.4, 29.5)	NA
	Hispanic	3.2 (1.7, 4.6)	-0.9 (-5.1, 3.2)	-0.5 (-6.2, 5.1)	3.4 (-10.3, 17.2)	-3.2 (-20.0, 13.6)	3.5 (-22.2, 29.1)	-25.3 (-75.8, 25.2)
	Other	-0.5 (-2.7, 1.8)	2.4 (-4.1, 9.0)	6.3 (-1.7, 14.2)	NA	3.1 (-16.9, 23.0)	-19.4 (-54.1, 15.3)	NA
	White	Ref	Ref	Ref	Ref	Ref	Ref	Ref
Education level								
	Some College	-0.1 (-1.5, 1.3)	3.2 (-0.5, 7.0)	1.1 (-4.4, 6.6)	4.8 (-7.2, 16.8)	-4.0 (-20.5, 12.4)	6.3 (-12.4, 24.9)	5.9 (-25.3, 37.2)
	College Graduate	2.4 (0.9, 3.8)	6.7 (2.9, 10.5)	5.7 (0.2, 11.2)	3.7 (-8.1, 15.5)	0.6 (-15.7, 16.8)	11.2 (-7.1, 29.5)	10.0 (-21.4, 41.5)
	High School/GED or less	Ref	Ref	Ref	Ref	Ref	Ref	Ref
Smoking								
	Past	-0.6 (-1.2, 0.0)	-0.3 (-1.9, 1.3)	0.5 (-1.7, 2.6)	-4.7 (-11.4, 2.1)	-2.6 (-7.9, 2.7)	-2.7 (-14.9, 9.5)	0.3 (-7.9, 8.4)
	Current	-11.2 (-12.9, -9.6)	-8.7 (-13.5, -3.9)	-7.1 (-13.4, -0.7)	-19.1 (-36.6, -1.7)	-17.3 (-45.5, 11.0)	-30.0 (-81.4, 21.5)	-22.6 (-48.7, 3.4)
	Never	Ref	Ref	Ref	Ref	Ref	Ref	Ref
Alcohol intake								
	Monthly	1.4 (0.5, 2.3)	3.1 (0.7, 5.5)	4.7 (1.4, 8.0)	1.5 (-7.3, 10.2)	1.6 (-6.4, 9.5)	-1.3 (-18.7, 16.2)	-4.3 (-16.2, 7.5)
	Weekly	3.6 (2.9, 4.3)	3.9 (1.9, 5.8)	4.2 (1.6, 6.8)	5.8 (-2.7, 14.2)	6.5 (0.2, 12.8)	-1.9 (-15.0, 11.2)	-5.4 (-15.1, 4.4)
	Daily	3.9 (2.9, 4.9)	5.1 (2.5, 7.6)	5.8 (2.5, 9.1)	4.8 (-5.3, 15.0)	8.8 (-0.5, 18.1)	10.0 (-7.1, 27.0)	-2.1 (-14.0, 9.7)
	Never or rarely	Ref	Ref	Ref	Ref	Ref	Ref	Ref
Current hormone therapy use								
	Yes	-1.5 (-2.6, -0.5)	-3.2 (-7.3, 0.8)	-2.0 (-10.1, 6.1)	-0.4 (-12.9, 12.2)	-3.2 (-13.6, 7.1)	3.7 (-19.2, 26.6)	12.6 (-4.7, 29.8)
	No	Ref	Ref	Ref	Ref	Ref	Ref	Ref
Self-rated health								
	Excellent	17.4 (15.7, 19.0)	15.0 (11.3, 18.8)	14.8 (9.6, 20.0)	20.6 (7.3, 33.8)	9.3 (-4.2, 22.7)	25.1 (3.7, 46.5)	8.1 (-45.5, 61.6)
	Very Good	11.4 (9.9, 13.0)	11.4 (8.1, 14.6)	11.9 (7.4, 16.4)	15.7 (3.7, 27.8)	5.7 (-6.1, 17.5)	11.1 (-5.0, 27.2)	0.6 (-52.2, 53.4)
	Good	6.2 (4.7, 7.8)	6.9 (3.7, 10.1)	7.5 (3.0, 12.0)	12.0 (0.3, 23.7)	3.1 (-8.2, 14.4)	-3.5 (-20.1, 13.2)	-3.6 (-56.2, 49.0)
	Fair/poor	Ref	Ref	Ref	Ref	Ref	Ref	Ref
Body mass index								
	Underweight	2.9 (0.7, 5.1)	1.5 (-4.2, 7.1)	3.6 (-4.6, 11.9)	6.1 (-13.3, 25.5)	15.1 (-3.3, 33.5)	-25.6 (-74.2, 23.0)	NA
	Overweight	-8.7 (-9.4, -8.1)	-8.9 (-10.7, -7.1)	-8.9 (-11.4, -6.5)	-0.5 (-8.7, 7.7)	-8.5 (-14.9, -2.0)	-7.7 (-19.4, 4.0)	-13.9 (-23.2, -4.6)
	Obese	-17.4 (-18.2, -16.6)	-16.6 (-18.8, -14.4)	-17.8 (-20.7, -14.8)	-7.8 (-16.7, 1.1)	-17.9 (-25.0, -10.9)	-15.3 (-30.8, 0.3)	-18.3 (-29.0, -7.6)
	Normal weight	Ref	Ref	Ref	Ref	Ref	Ref	Ref
History of cardiovascular disease								
	Yes	-2.8 (-4.0, -1.5)	-2.6 (-5.9, 0.7)	-1.3 (-5.8, 3.1)	0.9 (-14.5, 16.3)	-2.0 (-13.1, 9.1)	2.2 (-15.4, 19.7)	-9.2 (-36.1, 17.7)
	No	Ref	Ref	Ref	Ref	Ref	Ref	Ref
History of diabetes								
	Yes	-2.9 (-3.9, -2.0)	-5.4 (-7.7, -3.1)	-8.2 (-11.2, -5.1)	-7.6 (-16.2, 0.9)	-6.9 (-14.4, 0.6)	-6.8 (-27.5, 14.0)	6.0 (-10.1, 22.0)
	No	Ref	Ref	Ref	Ref	Ref	Ref	Ref
Accelerometer wear time		0.04 (0.04, 0.05)	0.05 (0.04, 0.06)	0.06 (0.04, 0.07)	0.08 (0.03, 0.13)	0.02 (-0.01, 0.06)	0.02 (-0.06, 0.10)	0.03 (-0.02, 0.08)
Time since cancer diagnosis		NA	-0.03 (-0.19, 0.13)	-0.05 (-0.25, 0.16)	-0.6 (-1.3, 0.1)	0.1 (-0.4, 0.6)	0.9 (-0.3, 2.1)	-0.3 (-1.1, 0.5)

Abbreviations: GED, general educational diploma; NA, not applicable; Ref, referent

Note: Any cancer does not include non-melanoma skin cancer. Models providing these estimates included age, race/ethnicity, education, smoking status, alcohol intake, menopausal hormone therapy use, general health, history of cardiovascular disease, history of diabetes, body mass index,

accelerometer wear time, and years since cancer diagnosis (except in the cancer-free model). Each column represents a separate fully adjusted statistical model with estimates and 95% confidence intervals.

Supplemental Table 5. Data from Supplemental Figure 2: Correlates (independent variables) of Time (min/day, dependent variable) at ≥40 steps/minute by Cancer Status and Type; the Women's Health Accelerometry Collaboration

Characteristic	*Level*	*Cancer-free*	*Any cancer*	*Breast cancer*	*Colon cancer*	*Endometrial cancer*	*Lung cancer*	*Melanoma skin cancer*
Age		-1.1 (-1.1, -1.1)	-1.1 (-1.2, -1.0)	-1.1 (-1.2, -0.9)	-1.3 (-1.7, -0.8)	-1.3 (-1.7, -0.9)	-1.3 (-2.1, -0.6)	-1.2 (-1.7, -0.7)
Race/ethnicity								
	Black	-2.8 (-3.8, -1.8)	-3.3 (-5.9, -0.8)	-2.9 (-6.4, 0.7)	-3.3 (-13.6, 7.1)	-11.9 (-26.2, 2.4)	11.3 (-6.8, 29.5)	NA
	Hispanic	1.0 (-0.3, 2.2)	-0.6 (-4.2, 3.0)	-1.0 (-5.7, 3.7)	6.1 (-6.6, 18.8)	-1.1 (-19.3, 17.2)	-0.1 (-23.0, 22.7)	-21.9 (-63.1, 19.4)
	Other	-0.9 (-2.9, 1.1)	5.0 (-0.9, 10.9)	9.3 (2.4, 16.2)	NA	-0.2 (-21.8, 21.5)	-13.3 (-43.6, 17.0)	NA
	White	Ref	Ref	Ref	Ref	Ref	Ref	Ref
Education level								
	Some College	-0.7 (-2.0, 0.6)	1.2 (-2.1, 4.5)	0.1 (-4.5, 4.8)	6.8 (-4.1, 17.8)	-10.6 (-28.4, 7.2)	8.1 (-8.7, 24.9)	-4.5 (-30.0, 21.0)
	College Graduate	2.0 (0.7, 3.3)	5.0 (1.7, 8.3)	4.7 (0.1, 9.4)	6.1 (-4.7, 16.9)	-3.5 (-21.1, 14.1)	12.7 (-3.7, 29.2)	0.3 (-25.4, 26.0)
	High School/GED or less	Ref	Ref	Ref	Ref	Ref	Ref	Ref
Smoking								
	Past	-0.6 (-1.2, 0.0)	-0.3 (-1.7, 1.2)	-0.3 (-2.1, 1.6)	-4.0 (-10.2, 2.1)	-2.6 (-8.4, 3.2)	-2.6 (-13.2, 8.0)	0.0 (-6.6, 6.7)
	Current	-9.3 (-10.8, -7.8)	-7.7 (-12.0, -3.4)	-6.9 (-12.5, -1.4)	-15.0 (-30.8, 0.9)	-10.4 (-41.1, 20.2)	-26.8 (-71.7, 18.0)	-15.0 (-36.2, 6.3)
	Never	Ref	Ref	Ref	Ref	Ref	Ref	Ref
Alcohol intake								
	Monthly	0.7 (-0.1, 1.5)	1.9 (-0.2, 4.0)	2.7 (-0.1, 5.5)	-0.6 (-8.6, 7.4)	3.0 (-5.6, 11.6)	1.5 (-13.8, 16.8)	-6.9 (-16.6, 2.8)
	Weekly	2.5 (1.9, 3.2)	3.1 (1.3, 4.8)	3.6 (1.4, 5.9)	5.4 (-2.2, 13.1)	4.5 (-2.3, 11.4)	0.4 (-11.0, 11.8)	-7.5 (-15.4, 0.5)
	Daily	2.5 (1.7, 3.4)	5.4 (3.2, 7.7)	5.6 (2.8, 8.4)	8.3 (-0.9, 17.5)	15.2 (5.1, 25.3)	9.7 (-5.2, 24.6)	-5.0 (-14.6, 4.7)
	Never or rarely	Ref	Ref	Ref	Ref	Ref	Ref	Ref
Current hormone therapy use								
	Yes	-1.7 (-2.6, -0.7)	-3.5 (-7.1, 0.1)	-1.0 (-8.0, 6.0)	-2.9 (-14.3, 8.4)	-8.6 (-19.9, 2.6)	5.1 (-14.9, 25.0)	7.3 (-6.8, 21.3)
	No	Ref	Ref	Ref	Ref	Ref	Ref	Ref
Self-rated health								
	Excellent	12.5 (11.0, 14.0)	11.3 (7.9, 14.6)	10.7 (6.2, 15.2)	14.0 (2.0, 26.0)	5.4 (-9.2, 20.0)	16.2 (-2.5, 34.8)	-5.3 (-49.0, 38.4)
	Very Good	6.6 (5.2, 8.0)	6.9 (4.0, 9.8)	7.3 (3.4, 11.2)	7.1 (-3.9, 18.0)	-1.9 (-14.7, 10.9)	9.3 (-4.7, 23.3)	-12.7 (-55.8, 30.4)
	Good	3.0 (1.6, 4.5)	3.2 (0.3, 6.1)	3.3 (-0.6, 7.1)	4.4 (-6.3, 15.0)	-1.9 (-14.1, 10.4)	-2.6 (-17.1, 12.0)	-16.9 (-59.9, 26.0)
	Fair/poor	Ref	Ref	Ref	Ref	Ref	Ref	Ref
Body mass index								
	Underweight	2.7 (0.7, 4.7)	0.2 (-4.8, 5.2)	2.2 (-4.9, 9.3)	8.5 (-8.9, 25.9)	6.4 (-13.6, 26.3)	-7.3 (-49.8, 35.2)	NA
	Overweight	-7.3 (-8.0, -6.7)	-7.9 (-9.5, -6.2)	-7.6 (-9.7, -5.5)	-1.0 (-8.3, 6.3)	-12.4 (-19.4, -5.4)	-5.1 (-15.3, 5.0)	-10.9 (-18.5, -3.3)
	Obese	-14.6 (-15.3, -13.8)	-13.8 (-15.7, -11.8)	-14.8 (-17.4, -12.3)	-7.9 (-15.8, 0.1)	-19.1 (-26.8, -11.4)	-9.5 (-22.9, 3.8)	-16.0 (-24.7, -7.2)
	Normal weight	Ref	Ref	Ref	Ref	Ref	Ref	Ref
History of cardiovascular disease								
	Yes	-1.9 (-3.0, -0.7)	-2.1 (-5.0, 0.8)	-2.0 (-5.9, 1.9)	3.5 (-10.5, 17.5)	-5.5 (-17.5, 6.6)	1.2 (-14.0, 16.4)	-10.5 (-32.5, 11.4)
	No	Ref	Ref	Ref	Ref	Ref	Ref	Ref
History of diabetes								
	Yes	-2.1 (-3.0, -1.3)	-3.3 (-5.4, -1.2)	-5.3 (-8.0, -2.7)	-5.8 (-13.6, 1.9)	-2.2 (-10.4, 6.0)	-4.2 (-22.3, 13.9)	2.6 (-10.5, 15.7)
	No	Ref	Ref	Ref	Ref	Ref	Ref	Ref
Accelerometer wear time		0.04 (0.03, 0.04)	0.04 (0.03, 0.05)	0.05 (0.03, 0.06)	0.05 (0.00, 0.09)	0.02 (-0.01, 0.06)	0.02 (-0.05, 0.09)	0.02 (-0.02, 0.06)
Time since cancer diagnosis		NA	-0.04 (-0.17, 0.10)	-0.1 (-0.3, 0.1)	-0.6 (-1.2, 0.0)	0.3 (-0.2, 0.8)	0.5 (-0.6, 1.5)	-0.4 (-1.0, 0.3)

Abbreviations: GED, general educational diploma; NA, not applicable; Ref, referent

Note: Any cancer does not include non-melanoma skin cancer. Models providing these estimates included age, race/ethnicity, education, smoking status, alcohol intake, menopausal hormone therapy use, general health, history of cardiovascular disease, history of diabetes, body mass index, accelerometer wear time, and years since cancer diagnosis (except in the cancer-free model). Each column represents a separate fully adjusted statistical model with estimates and 95% confidence intervals.

Supplemental Table 6: Correlates (independent variables) of Time in Moderate-to-Vigorous Physical Activity (min/day, dependent variable) by Cancer Status and Cohort; the Women's Health Accelerometry Collaboration

		WHS		WHI/OPACH	
Characteristic	*Level*	*Cancer-free*	*Any cancer*	*Cancer-free*	*Any cancer*
Age		-2.6 (-2.8, -2.5)	-2.7 (-3.0, -2.4)	-2.1 (-2.2, -1.9)	-1.8 (-2.2, -1.4)
Race/ethnicity					
	Black	-6.9 (-12.0, -1.7)	-22.0 (-40.6, -3.5)	-5.8 (-7.8, -3.7)	-3.1 (-8.5, 2.4)
	Hispanic	0.4 (-6.3, 7.0)	-8.7 (-29.2, 11.7)	2.6 (0.1, 5.0)	-1.1 (-7.8, 5.6)
	Other	-10.6 (-14.9, -6.3)	-2.6 (-15.0, 9.9)	NA	NA
	White	Ref	Ref	Ref	Ref
Education level					
	Some college	NA	NA	-1.1 (-3.3, 1.2)	-3.9 (-9.6, 1.7)
	College Graduate	-1.7 (-3.0, -0.4)	0.2 (-3.2, 3.7)	-2.9 (-5.1, -0.7)	2.7 (-3.0, 8.3)
	High School/GED or less	Ref	Ref	Ref	Ref
Smoking					
	Current	-17.0 (-20.5, -13.4)	-17.2 (-27.6, -6.8)	-13.0 (-18.0, -8.1)	-5.7 (-20.1, 8.7)
	Past	-0.8 (-2.1, 0.6)	-0.7 (-4.2, 2.8)	-2.4 (-4.1, -0.7)	1.9 (-2.6, 6.4)
	Never	Ref	Ref	Ref	Ref
Alcohol intake					
	Monthly	-0.03 (-2.34, 2.28)	-0.3 (-6.4, 5.8)	1.1 (-0.9, 3.0)	0.9 (-4.3, 6.1)
	Weekly	2.9 (1.3, 4.4)	2.6 (-1.4, 6.7)	6.1 (3.8, 8.3)	5.4 (-0.8, 11.6)
	Daily	2.0 (-0.1, 4.0)	3.1 (-2.1, 8.2)	5.2 (1.4, 9.0)	6.7 (-2.8, 16.2)
	Never or rarely	Ref	Ref	Ref	Ref
Current hormone therapy use					
	Yes	-4.7 (-6.8, -2.6)	-5.2 (-12.9, 2.6)	-2.3 (-7.1, 2.5)	9.0 (-29.6, 47.6)
	No	Ref	Ref	Ref	Ref
Self-rated health					
	Excellent	22.2 (17.6, 26.8)	13.0 (4.0, 22.0)	20.6 (16.9, 24.3)	20.9 (10.5, 31.3)
	Very Good	15.7 (11.2, 20.2)	6.7 (-1.5, 14.9)	12.0 (9.0, 14.9)	10.8 (3.8, 17.9)
	Good	9.4 (4.8, 13.9)	3.0 (-5.3, 11.2)	5.5 (2.6, 8.4)	3.5 (-3.1, 10.1)
	Fair/poor	Ref	Ref	Ref	Ref
Body mass index					
	Obese	-27.4 (-29.3, -25.5)	-28.1 (-33.0, -23.3)	-12.8 (-14.9, -10.6)	-13.2 (-19.1, -7.3)
	Overweight	-10.3 (-11.8, -8.8)	-10.8 (-14.7, -6.8)	-6.4 (-8.4, -4.4)	-9.0 (-14.4, -3.7)
	Underweight	3.3 (-1.4, 8.0)	0.1 (-11.5, 11.8)	5.9 (-1.0, 12.9)	5.5 (-13.5, 24.5)
	Normal weight	Ref	Ref	Ref	Ref
History of cardiovascular disease					
	Yes	-7.2 (-10.5, -3.9)	-12.4 (-20.5, -4.3)	-5.6 (-8.3, -3.0)	-7.7 (-14.6, -0.8)
	No	Ref	Ref	Ref	Ref
History of diabetes					
	Yes	-9.7 (-12.1, -7.3)	-15.3 (-21.1, -9.6)	-5.9 (-8.0, -3.9)	-6.7 (-11.7, -1.7)
	No	Ref	Ref	Ref	Ref
Accelerometer wear time		0.10 (0.09, 0.11)	0.12 (0.09, 0.14)	0.06 (0.05, 0.08)	0.08 (0.05, 0.11)

Abbreviations: GED, general educational diploma; NA, not applicable; Ref, referent

Note: Any cancer does not include non-melanoma skin cancer. Models providing these estimates included age, race/ethnicity, education, smoking status, alcohol intake, menopausal hormone therapy use, general health, history of cardiovascular disease, history of diabetes, body mass index, accelerometer wear time, and years since cancer diagnosis (except in the cancer-free model). Each column represents a separate fully adjusted statistical model with estimates and 95% confidence intervals.

Supplemental Table 7: Correlates (independent variables) of Average Vector Magnitude (VM, counts/min, dependent variable) by Cancer Status and Cohort; the Women's Health Accelerometry Collaboration

		WHS		WHI/OPACH	
Characteristic	*Level*	*Cancer-free*	*Any cancer*	*Cancer-free*	*Any cancer*
Age		-3.0 (-3.1, -2.9)	-2.9 (-3.3, -2.5)	-2.4 (-2.6, -2.3)	-2.1 (-2.6, -1.6)
Race/ethnicity					
	Black	-4.6 (-10.7, 1.4)	-19.5 (-41.5, 2.4)	-2.7 (-5.2, -0.2)	0.3 (-6.5, 7.2)
	Hispanic	2.5 (-5.3, 10.4)	-8.4 (-32.7, 15.9)	7.9 (4.9, 10.9)	4.2 (-4.2, 12.7)
	Other	-8.9 (-13.9, -3.9)	-1.1 (-15.9, 13.6)	NA	NA
	White	Ref	Ref	Ref	Ref
Education level					
	Some college	NA	NA	-4.1 (-6.9, -1.4)	-5.5 (-12.7, 1.7)
	College Graduate	-3.0 (-4.5, -1.4)	-0.4 (-4.5, 3.7)	-5.8 (-8.4, -3.1)	3.4 (-3.8, 10.6)
	High School/GED or less	Ref	Ref	Ref	Ref
Smoking					
	Current	-22.2 (-26.3, -18.1)	-21.2 (-33.5, -8.9)	-18.0 (-24.3, -11.7)	-14.6 (-33.0, 3.9)
	Past	-1.0 (-2.6, 0.5)	-1.8 (-5.9, 2.4)	-3.0 (-5.1, -0.9)	1.2 (-4.5, 6.9)
	Never	Ref	Ref	Ref	Ref
Alcohol intake					
	Monthly	-0.4 (-3.1, 2.4)	-1.2 (-8.4, 6.0)	1.3 (-1.1, 3.7)	4.4 (-2.2, 11.1)
	Weekly	3.1 (1.3, 5.0)	3.4 (-1.4, 8.2)	7.9 (5.1, 10.7)	8.3 (0.5, 16.2)
	Daily	3.4 (1.0, 5.8)	4.4 (-1.7, 10.6)	7.9 (3.2, 12.6)	10.4 (-1.7, 22.4)
	Never or rarely	Ref	Ref	Ref	Ref
Current hormone therapy use					
	Yes	-4.2 (-6.6, -1.7)	-7.5 (-16.6, 1.7)	-2.3 (-8.2, 3.6)	13.8 (-35.2, 62.8)
	No	Ref	Ref	Ref	Ref
Self-rated health					
	Excellent	24.8 (19.4, 30.2)	16.2 (5.6, 26.8)	25.1 (20.5, 29.7)	27.0 (13.9, 40.1)
	Very Good	17.4 (12.1, 22.6)	10.1 (0.4, 19.8)	16.1 (12.4, 19.8)	12.7 (3.9, 21.6)
	Good	10.5 (5.2, 15.9)	4.6 (-5.2, 14.3)	8.8 (5.2, 12.3)	5.7 (-2.6, 14.1)
	Fair/poor	Ref	Ref	Ref	Ref
Body mass index					
	Obese	-40.2 (-42.4, -38.0)	-41.2 (-47.0, -35.4)	-24.6 (-27.3, -22.0)	-24.7 (-32.2, -17.3)
	Overweight	-16.7 (-18.4, -14.9)	-16.4 (-21.1, -11.8)	-12.4 (-14.8, -9.9)	-13.3 (-20.2, -6.5)
	Underweight	6.8 (1.3, 12.3)	-0.9 (-14.7, 12.9)	10.0 (1.4, 18.6)	3.0 (-20.9, 26.8)
	Normal weight	Ref	Ref	Ref	Ref
History of cardiovascular disease					
	Yes	-7.9 (-11.7, -4.0)	-15.4 (-25.0, -5.8)	-7.3 (-10.6, -4.0)	-8.7 (-17.5, 0.0)
	No	Ref	Ref	Ref	Ref
History of diabetes					
	Yes	-12.4 (-15.2, -9.6)	-17.6 (-24.5, -10.8)	-8.3 (-10.8, -5.8)	-9.3 (-15.7, -2.9)
	No	Ref	Ref	Ref	Ref
Accelerometer wear time		-0.001 (-0.011, 0.009)	0.029 (0.001, 0.057)	0.008 (-0.005, 0.020)	0.04 (0.01, 0.08)

Abbreviations: GED, general educational diploma; NA, not applicable; Ref, referent

Note: Any cancer does not include non-melanoma skin cancer. Models providing these estimates included age, race/ethnicity, education, smoking status, alcohol intake, menopausal hormone therapy use, general health, history of cardiovascular disease, history of diabetes, body mass index, accelerometer wear time, and years since cancer diagnosis (except in the cancer-free model). Each column represents a separate fully adjusted statistical model with estimates and 95% confidence intervals.

Supplemental Table 8: Correlates (independent variables) of Total Step Volume (steps/day, dependent variable) by Cancer Status and Cohort; the Women's Health Accelerometry Collaboration

		WHS		WHI/OPACH	
Characteristic	*Level*	*Cancer-free*	*Any cancer*	*Cancer-free*	*Any cancer*
Age		-157.9 (-164.6, -151.3)	-158.7 (-175.6, -141.8)	-130.0 (-138.3, -121.7)	-104.1 (-126.1, -82.1)
Race/ethnicity					
	Black	-278.6 (-569.0, 11.8)	-942.3 (-1982.1, 97.5)	-313.1 (-433.4, -192.7)	-83.0 (-386.7, 220.6)
	Hispanic	433.9 (55.7, 812.0)	-641.1 (-1790.0, 507.9)	433.2 (290.4, 576.0)	412.0 (37.1, 786.9)
	Other	-178.3 (-419.4, 62.9)	210.1 (-489.0, 909.1)	NA	NA
	White	Ref	Ref	Ref	Ref
Education level					
	Some college	NA	NA	20.0 (-109.9, 149.8)	18.8 (-299.4, 336.9)
	College Graduate	79.6 (4.0, 155.2)	339.0 (144.7, 533.4)	-193.7 (-321.2, -66.2)	315.2 (-3.2, 633.5)
	High School/GED or less	Ref	Ref	Ref	Ref
Smoking					
	Current	-1217.5 (-1416.4, -1018.6)	-1014.4 (-1597.5, -431.3)	-934.1 (-1224.5, -643.8)	-711.2 (-1506.1, 83.7)
	Past	-40.6 (-116.7, 35.4)	-45.4 (-243.1, 152.4)	-147.1 (-247.1, -47.1)	76.7 (-174.0, 327.3)
	Never	Ref	Ref	Ref	Ref
Alcohol intake					
	Monthly	28.9 (-101.6, 159.3)	159.2 (-182.6, 501.0)	143.1 (27.1, 259.2)	257.1 (-29.6, 543.8)
	Weekly	252.8 (165.8, 339.8)	289.6 (61.0, 518.3)	460.1 (327.0, 593.2)	475.9 (129.0, 822.8)
	Daily	256.3 (142.1, 370.6)	479.4 (188.6, 770.2)	478.0 (250.3, 705.8)	810.7 (280.6, 1340.8)
	Never or rarely	Ref	Ref	Ref	Ref
Current hormone therapy use					
	Yes	-208.0 (-326.9, -89.2)	-443.7 (-878.2, -9.3)	-73.1 (-355.4, 209.2)	1372.1 (-792.2, 3536.4)
	No	Ref	Ref	Ref	
Self-rated health					
	Excellent	1616.6 (1355.4, 1877.9)	1246.5 (744.3, 1748.7)	1624.2 (1406.5, 1841.9)	1831.6 (1250.2, 2413.0)
	Very Good	1047.5 (794.4, 1300.6)	852.1 (393.3, 1310.8)	969.1 (793.7, 1144.5)	956.5 (562.6, 1350.3)
	Good	610.3 (353.8, 866.8)	483.2 (20.4, 945.9)	462.8 (291.7, 633.8)	482.3 (112.2, 852.4)
	Fair/poor	Ref	Ref	Ref	Ref
Body mass index					
	Obese	-1797.5 (-1902.8, -1692.1)	-1768.8 (-2043.1, -1494.5)	-1047.7 (-1174.2, -921.2)	-863.9 (-1197.6, -530.2)
	Overweight	-827.4 (-911.4, -743.4)	-811.6 (-1032.5, -590.7)	-507.0 (-624.2, -389.7)	-591.8 (-894.7, -288.9)
	Underweight	284.7 (18.4, 551.0)	-67.3 (-721.6, 587.1)	430.5 (12.4, 848.6)	-202.5 (-1259.5, 854.5)
	Normal weight	Ref	Ref	Ref	Ref
History of cardiovascular disease					
	Yes	-351.4 (-535.8, -166.9)	-631.8 (-1087.6, -176.1)	-311.7 (-469.6, -153.8)	-347.1 (-735.3, 41.2)
	No	Ref	Ref	Ref	Ref
History of diabetes					
	Yes	-396.5 (-531.0, -261.9)	-607.7 (-930.4, -284.9)	-344.4 (-464.8, -223.9)	-445.3 (-727.9, -162.7)
	No	Ref	Ref	Ref	Ref
Accelerometer wear time		6.4 (5.9, 6.9)	6.9 (5.6, 8.2)	5.0 (4.4, 5.6)	6.0 (4.4, 7.6)

Abbreviations: GED, general educational diploma; NA, not applicable; Ref, referent

Note: Any cancer does not include non-melanoma skin cancer. Models providing these estimates included age, race/ethnicity, education, smoking status, alcohol intake, menopausal hormone therapy use, general health, history of cardiovascular disease, history of diabetes, body mass index, accelerometer wear time, and years since cancer diagnosis (except in the cancer-free model). Each column represents a separate fully adjusted statistical model with estimates and 95% confidence intervals.

Supplemental Table 9: Correlates (independent variables) of Peak 30-minute Cadence (steps/min, dependent variable) by Cancer Status and Cohort; the Women's Health Accelerometry Collaboration

		WHS		WHI/OPACH	
Characteristic	***Level***	***Cancer-free***	***Any cancer***	***Cancer-free***	***Any cancer***
Age		-1.3 (-1.3, -1.2)	-1.4 (-1.5, -1.2)	-1.2 (-1.3, -1.1)	-0.9 (-1.2, -0.7)
Race/ethnicity		Ref	Ref	Ref	Ref
	Black	-2.3 (-5.1, 0.5)	-8.7 (-19.0, 1.5)	-2.9 (-4.2, -1.6)	-2.2 (-5.6, 1.1)
	Hispanic	2.2 (-1.4, 5.8)	-7.5 (-18.8, 3.8)	3.7 (2.1, 5.2)	2.6 (-1.5, 6.7)
	Other	-0.6 (-2.9, 1.7)	1.7 (-5.2, 8.6)	NA	NA
	White	Ref	Ref	Ref	Ref
Education level					
	Some college	NA	NA	2.5 (1.1, 3.9)	4.1 (0.6, 7.6)
	College Graduate	2.4 (1.7, 3.1)	4.7 (2.8, 6.6)	-0.3 (-1.7, 1.0)	5.3 (1.8, 8.8)
	High School/GED or less	Ref	Ref	Ref	Ref
Smoking					
	Current	-11.8 (-13.7, -9.9)	-9.0 (-14.8, -3.3)	-9.7 (-12.8, -6.5)	-6.6 (-15.2, 1.9)
	Past	-0.2 (-0.9, 0.6)	-0.8 (-2.7, 1.2)	-1.7 (-2.9, -0.6)	1.7 (-1.1, 4.6)
	Never	Ref	Ref	Ref	Ref
Alcohol intake					
	Monthly	1.249 (-0.004, 2.503)	3.9 (0.5, 7.2)	1.9 (0.6, 3.1)	2.0 (-1.2, 5.2)
	Weekly	3.2 (2.4, 4.1)	3.6 (1.4, 5.9)	4.9 (3.4, 6.3)	3.7 (-0.2, 7.5)
	Daily	3.5 (2.4, 4.6)	4.7 (1.9, 7.6)	5.0 (2.5, 7.4)	4.8 (-1.2, 10.7)
	Never or rarely	Ref	Ref	Ref	Ref
Current hormone therapy use					
	Yes	-1.7 (-2.8, -0.6)	-4.2 (-8.5, 0.0)	-1.0 (-4.0, 2.1)	22.2 (-1.8, 46.2)
	No	Ref	Ref	Ref	Ref
Self-rated health					
	Excellent	17.1 (14.6, 19.6)	14.1 (9.2, 19.1)	18.8 (16.5, 21.2)	19.4 (13.0, 25.8)
	Very Good	11.4 (9.0, 13.8)	11.2 (6.7, 15.7)	11.8 (9.9, 13.7)	12.3 (7.9, 16.6)
	Good	6.8 (4.3, 9.2)	7.5 (2.9, 12.1)	5.8 (3.9, 7.6)	6.1 (2.0, 10.2)
	Fair/poor	Ref	Ref	Ref	Ref
Body mass index					
	Obese	-19.1 (-20.1, -18.1)	-19.1 (-21.8, -16.4)	-13.4 (-14.7, -12.0)	-10.0 (-13.7, -6.3)
	Overweight	-9.4 (-10.2, -8.6)	-8.9 (-11.1, -6.8)	-6.5 (-7.7, -5.2)	-7.2 (-10.6, -3.9)
	Underweight	3.3 (0.7, 5.9)	1.3 (-5.2, 7.7)	1.7 (-2.8, 6.2)	2.6 (-9.3, 14.5)
	Normal weight	Ref	Ref	Ref	Ref
History of cardiovascular disease					
	Yes	-3.2 (-4.9, -1.4)	-3.7 (-8.2, 0.8)	-2.3 (-4.0, -0.6)	-2.2 (-6.5, 2.1)
	No	Ref	Ref	Ref	Ref
History of diabetes					
	Yes	-3.0 (-4.3, -1.7)	-6.2 (-9.4, -3.0)	-2.7 (-4.0, -1.4)	-4.2 (-7.3, -1.1)
	No	Ref	Ref	Ref	Ref
Accelerometer wear time		0.04 (0.04, 0.05)	0.05 (0.04, 0.06)	0.04 (0.03, 0.04)	0.05 (0.03, 0.07)

Abbreviations: GED, general educational diploma; NA, not applicable; Ref, referent

Note: Any cancer does not include non-melanoma skin cancer. Models providing these estimates included age, race/ethnicity, education, smoking status, alcohol intake, menopausal hormone therapy use, general health, history of cardiovascular disease, history of diabetes, body mass index, accelerometer wear time, and years since cancer diagnosis (except in the cancer-free model). Each column represents a separate fully adjusted statistical model with estimates and 95% confidence intervals.

Supplemental Table 10: Correlates (independent variables) of Time (min/day, dependent variable) at ≥40 steps/minute by Cancer Status and Cohort; the Women's Health Accelerometry Collaboration

		WHS		WHI/OPACH	
Characteristic	*Level*	*Cancer-free*	*Any cancer*	*Cancer-free*	*Any cancer*
Age		-1.2 (-1.3, -1.1)	-1.2 (-1.3, -1.0)	-0.8 (-0.9, -0.8)	-0.6 (-0.8, -0.5)
Race/ethnicity		Ref	Ref	Ref	Ref
	Black	-1.4 (-4.0, 1.3)	-7.2 (-16.6, 2.2)	-2.0 (-3.0, -1.0)	-1.0 (-3.6, 1.5)
	Hispanic	2.1 (-1.3, 5.5)	-7.2 (-17.6, 3.1)	2.5 (1.3, 3.7)	3.3 (0.1, 6.5)
	Other	-1.1 (-3.3, 1.1)	4.3 (-2.0, 10.7)	NA	NA
	White	Ref	Ref	Ref	Ref
Education level					
	Some college	NA	NA	2.3 (1.2, 3.4)	2.8 (0.1, 5.5)
	College Graduate	2.5 (1.8, 3.2)	5.0 (3.2, 6.7)	-0.5 (-1.5, 0.6)	3.4 (0.7, 6.2)
	High School/GED or less	Ref	Ref	Ref	Ref
Smoking					
	Current	-10.2 (-12.0, -8.4)	-8.5 (-13.7, -3.2)	-6.3 (-8.7, -3.8)	-3.6 (-10.1, 2.9)
	Past	-0.4 (-1.1, 0.3)	-0.2 (-2.0, 1.6)	-1.0 (-1.8, -0.1)	0.4 (-1.7, 2.5)
	Never	Ref	Ref	Ref	Ref
Alcohol intake					
	Monthly	0.5 (-0.7, 1.6)	2.1 (-1.0, 5.1)	1.1 (0.2, 2.1)	1.6 (-0.9, 4.0)
	Weekly	2.4 (1.6, 3.2)	2.8 (0.7, 4.8)	3.1 (2.0, 4.2)	3.0 (0.1, 6.0)
	Daily	2.3 (1.3, 3.3)	4.7 (2.1, 7.3)	3.0 (1.1, 5.0)	7.4 (2.8, 12.0)
	Never or rarely	Ref	Ref	Ref	Ref
Current hormone therapy use					
	Yes	-1.9 (-3.0, -0.8)	-4.3 (-8.2, -0.4)	-0.7 (-3.1, 1.6)	14.6 (-3.8, 33.1)
	No	Ref	Ref	Ref	Ref
Self-rated health					
	Excellent	13.3 (11.0, 15.7)	11.2 (6.7, 15.8)	12.9 (11.0, 14.7)	12.8 (7.8, 17.7)
	Very Good	7.7 (5.4, 10.0)	7.5 (3.3, 11.6)	6.6 (5.1, 8.1)	6.8 (3.5, 10.2)
	Good	4.4 (2.1, 6.7)	4.0 (-0.1, 8.2)	2.6 (1.2, 4.0)	2.7 (-0.5, 5.8)
	Fair/poor	Ref	Ref	Ref	Ref
Body mass index					
	Obese	-16.5 (-17.4, -15.5)	-16.2 (-18.7, -13.7)	-9.8 (-10.9, -8.8)	-7.3 (-10.2, -4.5)
	Overweight	-8.0 (-8.8, -7.2)	-8.1 (-10.1, -6.1)	-4.9 (-5.9, -3.9)	-5.7 (-8.2, -3.2)
	Underweight	2.6 (0.2, 5.0)	0.5 (-5.4, 6.4)	3.8 (0.2, 7.4)	-1.2 (-10.1, 7.7)
	Normal weight	Ref	Ref	Ref	Ref
History of cardiovascular disease					
	Yes	-2.6 (-4.3, -1.0)	-3.7 (-7.9, 0.4)	-1.2 (-2.5, 0.2)	-1.2 (-4.5, 2.1)
	No	Ref	Ref	Ref	Ref
History of diabetes					
	Yes	-2.3 (-3.5, -1.1)	-3.8 (-6.7, -0.9)	-1.8 (-2.8, -0.8)	-2.4 (-4.8, 0.0)
	No	Ref	Ref	Ref	Ref
Accelerometer wear time		0.04 (0.04, 0.05)	0.04 (0.03, 0.05)	0.02 (0.02, 0.03)	0.03 (0.01, 0.04)

Abbreviations: GED, general educational diploma; NA, not applicable; Ref, referent

Note: Any cancer does not include non-melanoma skin cancer. Models providing these estimates included age, race/ethnicity, education, smoking status, alcohol intake, menopausal hormone therapy use, general health, history of cardiovascular disease, history of diabetes, body mass index, accelerometer wear time, and years since cancer diagnosis (except in the cancer-free model). Each column represents a separate fully adjusted statistical model with estimates and 95% confidence intervals.

Supplemental Table 11. Data from Figure 4: Correlates (independent variables) of Sedentary Behavior (min/day, dependent variable) by Cancer Status and Type; the Women's Health Accelerometry Collaboration

Characteristic	*Level*	*Cancer-free*	*Any cancer*	*Breast cancer*	*Colon cancer*	*Endometrial cancer*	*Lung cancer*	*Melanoma skin cancer*
Age		3.6 (3.4, 3.8)	3.0 (2.4, 3.5)	2.9 (2.1, 3.7)	2.3 (0.1, 4.6)	0.9 (-0.8, 2.6)	2.6 (-1.1, 6.3)	2.9 (0.8, 5.1)
Race/ethnicity								
	Black	-16.2 (-20.8, -11.7)	-20.3 (-33.6, -7.0)	-20.3 (-38.7, -2.0)	-40.2 (-89.5, 9.1)	-11.6 (-68.5, 45.3)	2.4 (-79.7, 84.4)	NA
	Hispanic	-22.6 (-28.3, -17.0)	-20.9 (-38.1, -3.7)	-18.4 (-41.9, 5.2)	-43.2 (-102.8, 16.4)	-25.6 (-94.9, 43.8)	-124.4 (-228.0, -20.9)	-110.7 (-279.1, 57.7)
	Other	-0.4 (-9.1, 8.3)	-8.2 (-34.8, 18.4)	-14.0 (-47.4, 19.4)	NA	-40.0 (-120.7, 40.7)	13.1 (-121.3, 147.4)	NA
	White	Ref	Ref	Ref	Ref	Ref	Ref	Ref
Education level								
	Some College	12.5 (6.9, 18.0)	-0.5 (-16.0, 15.0)	-2.3 (-25.3, 20.7)	11.5 (-40.0, 63.0)	21.4 (-47.2, 90.1)	-39.7 (-115.5, 36.1)	33.2 (-70.8, 137.3)
	College Graduate	16.4 (10.8, 22.0)	7.1 (-8.4, 22.6)	1.7 (-21.3, 24.6)	1.5 (-49.2, 52.3)	22.3 (-45.0, 89.6)	-24.7 (-98.6, 49.1)	45.6 (-59.3, 150.5)
	High School/GED or less	Ref	Ref	Ref	Ref	Ref	Ref	Ref
Smoking								
	Past	3.0 (0.6, 5.4)	6.4 (-0.2, 12.9)	6.3 (-2.8, 15.4)	18.0 (-11.0, 47.0)	13.4 (-8.3, 35.1)	12.2 (-34.9, 59.2)	11.8 (-15.3, 38.9)
	Current	34.6 (28.1, 41.1)	37.5 (17.9, 57.2)	30.1 (3.6, 56.6)	17.9 (-56.8, 92.5)	91.8 (-22.7, 206.2)	-26.7 (-224.8, 171.5)	23.6 (-63.3, 110.4)
	Never	Ref	Ref	Ref	Ref	Ref	Ref	Ref
Alcohol intake								
	Monthly	0.8 (-2.7, 4.3)	-8.0 (-17.7, 1.6)	-9.1 (-22.7, 4.6)	-6.3 (-43.8, 31.2)	-13.0 (-45.9, 20.0)	-56.1 (-123.9, 11.7)	1.8 (-37.5, 41.2)
	Weekly	-5.7 (-8.5, -2.9)	-12.4 (-20.3, -4.5)	-9.2 (-20.0, 1.6)	-26.2 (-62.1, 9.7)	-21.4 (-47.1, 4.3)	-23.0 (-73.3, 27.3)	4.0 (-28.3, 36.4)
	Daily	-9.0 (-12.8, -5.1)	-12.3 (-22.5, -2.1)	-10.7 (-24.3, 3.0)	-46.2 (-89.4, -2.9)	-54.8 (-92.5, -17.1)	-30.8 (-96.6, 34.9)	11.4 (-28.0, 50.8)
	Never or rarely	Ref	Ref	Ref	Ref	Ref	Ref	Ref
Current hormone therapy use								
	Yes	-0.2 (-4.3, 3.8)	11.2 (-5.2, 27.6)	2.5 (-31.4, 36.3)	3.1 (-50.4, 56.5)	9.1 (-32.9, 51.0)	75.3 (-13.0, 163.6)	-10.5 (-68.0, 46.9)
	No	Ref	Ref	Ref	Ref	Ref	Ref	Ref
Self-rated health								
	Excellent	-33.4 (-39.8, -26.9)	-35.3 (-50.4, -20.1)	-39.6 (-61.4, -17.8)	-40.8 (-97.2, 15.6)	-2.1 (-56.7, 52.6)	-4.8 (-87.3, 77.6)	-38.6 (-216.9, 139.8)
	Very Good	-25.2 (-31.3, -19.2)	-24.6 (-37.8, -11.4)	-22.6 (-41.4, -3.9)	-50.7 (-102.0, 0.5)	-7.5 (-55.5, 40.5)	-37.6 (-99.6, 24.3)	-13.7 (-189.6, 162.1)
	Good	-16.6 (-22.6, -10.5)	-14.0 (-27.0, -0.9)	-7.7 (-26.4, 11.0)	-46.2 (-96.2, 3.7)	6.2 (-39.8, 52.2)	10.2 (-54.2, 74.6)	8.7 (-166.4, 183.9)
	Fair/poor	Ref	Ref	Ref	Ref	Ref	Ref	Ref
Body mass index								
	Underweight	-20.0 (-28.7, -11.4)	-3.0 (-25.7, 19.7)	5.2 (-29.3, 39.6)	51.2 (-29.8, 132.2)	4.9 (-69.8, 79.5)	23.5 (-165.1, 212.0)	NA
	Overweight	36.8 (34.1, 39.4)	33.2 (25.8, 40.7)	33.1 (22.9, 43.2)	29.2 (-4.7, 63.1)	27.5 (1.5, 53.6)	68.4 (23.6, 113.3)	23.9 (-7.2, 54.9)
	Obese	75.6 (72.4, 78.8)	75.9 (67.0, 84.7)	85.0 (72.6, 97.3)	28.2 (-8.8, 65.2)	88.4 (59.6, 117.3)	62.7 (3.5, 121.9)	73.4 (37.8, 109.1)
	Normal weight	Ref	Ref	Ref	Ref	Ref	Ref	Ref
History of cardiovascular disease								
	Yes	12.0 (7.1, 17.0)	13.8 (0.6, 27.0)	9.2 (-9.5, 27.8)	-2.6 (-68.2, 63.0)	17.9 (-27.1, 62.9)	16.5 (-51.0, 84.0)	11.7 (-77.8, 101.3)
	No	Ref	Ref	Ref	Ref	Ref	Ref	Ref
History of diabetes								
	Yes	17.9 (14.2, 21.6)	21.1 (11.6, 30.5)	27.2 (14.5, 39.9)	26.3 (-10.2, 62.8)	22.9 (-7.5, 53.3)	-16.9 (-96.9, 63.1)	36.9 (-16.5, 90.4)
	No	Ref	Ref	Ref	Ref	Ref	Ref	Ref
Accelerometer wear time		0.6 (0.6, 0.6)	0.6 (0.5, 0.6)	0.6 (0.5, 0.6)	0.5 (0.3, 0.7)	0.6 (0.4, 0.7)	0.5 (0.2, 0.8)	0.5 (0.4, 0.7)
Time since cancer diagnosis		NA	-0.2 (-0.8, 0.5)	-0.5 (-1.3, 0.4)	3.4 (0.5, 6.2)	0.3 (-1.8, 2.3)	-3.3 (-7.9, 1.3)	1.8 (-0.8, 4.5)

Abbreviations: GED, general educational diploma; NA, not applicable; Ref, referent

Note: Any cancer does not include non-melanoma skin cancer. Models providing these estimates included age, race/ethnicity, education, smoking status, alcohol intake, menopausal hormone therapy use, general health, history of cardiovascular disease, history of diabetes, body mass index,

accelerometer wear time, and years since cancer diagnosis (except in the cancer-free model). Each column represents a separate fully adjusted statistical model with estimates and 95% confidence intervals.

Supplemental Table 12: Correlates (independent variables) of Sedentary Behavior (min/day, dependent variable) by Cancer Status and Cohort; the Women's Health Accelerometry Collaboration

		WHS		WHI/OPACH	
Characteristic	*Level*	*Cancer-free*	*Any cancer*	*Cancer-free*	*Any cancer*
Age		3.6 (3.3, 3.8)	3.0 (2.3, 3.6)	3.6 (3.3, 4.0)	2.7 (1.5, 3.9)
Race/ethnicity					
	Black	-10.5 (-21.1, 0.0)	4.1 (-35.7, 43.8)	-17.2 (-22.8, -11.5)	-24.6 (-40.6, -8.6)
	Hispanic	-13.8 (-27.5, 0.0)	-20.9 (-64.8, 23.0)	-25.1 (-31.8, -18.5)	-23.6 (-43.4, -3.9)
	Other	0.2 (-8.5, 9.0)	-6.4 (-33.2, 20.3)	NA	NA
	White	Ref	Ref	Ref	Ref
Education level					
	Some college	NA	NA	14.0 (7.9, 20.0)	12.5 (-4.3, 29.4)
	College Graduate	5.7 (2.9, 8.4)	4.5 (-2.9, 11.9)	16.4 (10.5, 22.4)	-4.8 (-21.6, 12.0)
	High School/GED or less	Ref	Ref	Ref	Ref
Smoking					
	Current	33.5 (26.2, 40.7)	35.2 (12.9, 57.5)	39.5 (25.0, 54.0)	45.2 (3.4, 87.1)
	Past	2.4 (-0.4, 5.2)	7.9 (0.4, 15.5)	5.2 (0.4, 9.9)	0.7 (-12.5, 13.8)
	Never	Ref	Ref	Ref	Ref
Alcohol intake					
	Monthly	0.9 (-3.9, 5.6)	2.9 (-10.1, 16.0)	-1.3 (-6.7, 4.1)	-20.8 (-36.0, -5.6)
	Weekly	-3.5 (-6.6, -0.3)	-7.9 (-16.6, 0.9)	-14.2 (-20.5, -8.0)	-25.7 (-44.1, -7.2)
	Daily	-6.9 (-11.1, -2.8)	-8.5 (-19.6, 2.6)	-17.3 (-27.8, -6.8)	-18.9 (-46.8, 9.1)
	Never or rarely	Ref	Ref	Ref	Ref
Current hormone therapy use					
	Yes	0.0 (-4.3, 4.3)	13.7 (-2.9, 30.3)	0.8 (-12.4, 13.9)	-24.2 (-138.3, 90.0)
	No	Ref	Ref	Ref	Ref
Self-rated health					
	Excellent	-30.5 (-40.0, -21.0)	-29.7 (-48.9, -10.5)	-36.3 (-46.4, -26.1)	-58.2 (-88.8, -27.6)
	Very Good	-22.9 (-32.1, -13.7)	-23.6 (-41.1, -6.0)	-26.3 (-34.5, -18.1)	-21.3 (-42.0, -0.6)
	Good	-14.4 (-23.7, -5.1)	-11.3 (-29.0, 6.4)	-18.2 (-26.2, -10.2)	-16.7 (-36.3, 2.8)
	Fair/poor	Ref	Ref	Ref	Ref
Body mass index					
	Obese	80.0 (76.2, 83.8)	82.0 (71.5, 92.5)	64.8 (58.9, 70.7)	59.4 (42.0, 76.8)
	Overweight	37.7 (34.6, 40.7)	34.7 (26.2, 43.1)	32.7 (27.2, 38.2)	26.1 (10.2, 42.0)
	Underweight	-19.4 (-29.1, -9.7)	1.4 (-23.6, 26.4)	-22.0 (-41.5, -2.4)	-11.6 (-68.1, 45.0)
	Normal weight	Ref	Ref	Ref	Ref
History of cardiovascular disease					
	Yes	10.1 (3.4, 16.8)	16.6 (-0.8, 34.0)	14.4 (7.0, 21.7)	11.4 (-9.0, 31.9)
	No	Ref	Ref	Ref	Ref
History of diabetes					
	Yes	19.1 (14.2, 24.0)	25.1 (12.8, 37.5)	15.9 (10.2, 21.5)	15.3 (0.4, 30.2)
	No	Ref	Ref	Ref	Ref
Accelerometer wear time		0.6 (0.6, 0.6)	0.6 (0.5, 0.6)	0.6 (0.6, 0.7)	0.5 (0.4, 0.6)

Abbreviations: GED, general educational diploma; NA, not applicable; Ref, referent

Note: Any cancer does not include non-melanoma skin cancer. Models providing these estimates included age, race/ethnicity, education, smoking status, alcohol intake, menopausal hormone therapy use, general health, history of cardiovascular disease, history of diabetes, body mass index, accelerometer wear time, and years since cancer diagnosis (except in the cancer-free model). Each column represents a separate fully adjusted statistical model with estimates and 95% confidence intervals.
